# The genome sequencing and comparative analysis of a wild kiwifruit *Actinidia eriantha*

**DOI:** 10.1186/s43897-022-00034-z

**Published:** 2022-05-08

**Authors:** Xiaohong Yao, Shuaibin Wang, Zupeng Wang, Dawei Li, Quan Jiang, Qiong Zhang, Lei Gao, Caihong Zhong, Hongwen Huang, Yifei Liu

**Affiliations:** 1 Key Laboratory of Plant Germplasm Enhancement and Specially Agriculture, Wuhan Botanical Garden, the Chinese Academy of Sciences, Wuhan, 430074 China; 2 Department of Bioinformatics, College of Life Sciences, Zhejiang University, Hangzhou, 310058 China; 3 College of Life Sciences, University of Chinese Academy of Sciences, Beijing, 100049 China; 4 Lushan Botanical Garden, Chinese Academy of Sciences, Jiujiang, 332900 China; 5 College of Pharmacy, Hubei University of Chinese Medicine, Wuhan, 430065 China

**Keywords:** Kiwifruit, Genome, Evolution, Vitamin C, Disease resistance, Introgression breeding

## Abstract

The current kiwifruit industry is mainly based on the cultivars derived from the species *Actinidia chinensis* (Ac) which may bring risks such as canker disease. Introgression of desired traits from wild relatives is an important method for improving kiwifruit cultivars. *Actinidia eriantha* (Ae) is a particularly important taxon used for hybridization or introgressive breeding of new kiwifruit cultivars because of its valued species-specific traits. Here, we assembled a chromosome-scale high-quality genome of a Ae sample which was directly collected from its wild populations. Our analysis revealed that 41.3% of the genome consists of repetitive elements, comparable to the percentage in Ac and Ae cultivar “White” genomes. The genomic structural variation, including the presence/absence-variation (PAV) of genes, is distinct between Ae and Ac, despite both sharing the same two kiwifruit-specific whole genome duplication (WGD) events. This suggests that a post-WGD divergence mechanism occurred during their evolution. We further investigated genes involved in ascorbic acid biosynthesis and disease-resistance of Ae, and we found introgressive genome could contribute to the complex relationship between Ae and other representative kiwifruit taxa. Collectively, the Ae genome offers valuable genetic resource to accelerate kiwifruit breeding applications.

**Supplementary Information:**

The online version contains supplementary material available at 10.1186/s43897-022-00034-z.

## Core

*Actinidia eriantha* is a valued wild *Actinidia* taxon for introgressive breeding of kiwifruit cultivars. The genome assembly of a wild *A. eriantha* plant provided insights into genome evolution and structural variations between *A. eriantha* and one of the cultivated kiwifruits *A. chinensis*. The genes involved in ascorbic acid biosynthesis and disease-resistance were also potentially different between them, and introgressive genome could contribute to the complex relationship between *A. eriantha* and other representative kiwifruit taxa.

## Gene & Accession Numbers

The raw sequence reads and genome assembly have been deposited in NCBI database under BioProject accession PRJNA721303. All raw reads in the Sequence Read Archive (SRA) can be accessed under accessions SRR14212090-SRR14212115 and SRR15012233-SRR15012260.

## Introduction

Kiwifruit is one of the best examples of the successful domestication and commercialization of a fruit crop in the twentieth century (Ferguson 2016). Since 1904 the first kiwifruit, *Actinidia chinensis* var. *deliciosa* from wild populations of Yichang, China, was introduced into New Zealand, its initial domestication and the subsequent development of commercial cultivation made it increasingly important for the global fresh fruit market (Huang 2014). Particularly, over the past two decades, kiwifruit is the fastest growing crop in per capita production of major fruit groups (FAO Statistics; http://www.fao.org/statistics/en/). This is likely to grow continuously in the next decade as the kiwifruit industry addresses the *Pseudomonas syringae* pv. *actinidiae* (Psa) epidemic, the main kiwifruit bacterial disease (Belrose Inc. 2016). The wide demands of diversified and resistant germplasm feeding the world kiwifruit industry has prompted its substantial acceleration recently by a combination of diverse strategies. Among these are hybridization or introgression breeding, which tends to bring desired traits from wild relatives into kiwifruit cultivars (Huang and Liu 2014).

*Actinidia eriantha* Bentham (hereafter, Ae), which is known as “Maohua” kiwifruit in Chinese, is one of the most valued wild *Actinidia* resources used for introgression breeding of kiwifruit cultivars. Currently, many kiwifruit hybrid cultivars are crossed and domesticated between Ae and *A. chinensis* Planchon (hereafter, Ac), in which the latter is traditionally selected for breeding of nearly all cultivated kiwifruits. Although Ae is taxonomically and phylogenetically distinct from Ac, crossing between them is easily conducted (Liu et al. 2017). Ae is characterized by several species-specific traits, such as the exceptionally high content of vitamin C in its matured fruits (500–1379 mg per 100 g fresh weight, compared to 50–420 mg in Ac) and the potentially high resistance to Psa disease (Huang et al. 2004; Wang et al. 2017). The milky white hairs densely covering on the surface of fruits and stems of Ae are also specific in the *Actinidia* genus. Understanding the genome and evolution underlying trait variations of Ae is thus important for kiwifruit breeding applications.

The *Actinidia* genus belongs to the Actinidiaceae family within the Ericales order, which is an early diverging lineage in the asterids. Genomic analyses indicates that both common and lineage-specific whole-genome duplication events (WGDs) have occurred across different lineages of asterids. Ac shows an ancient triplication event (the γ event shared by core eudicots) and two recently kiwifruit-specific WGD events (Ad-α and Ad-β) (Huang et al. 2013). A recent reanalysis of the genome further showed evidence that both kiwifruit-specific WGD events were tetraploidization events, with Ad-α occurring ~ 18–20 million years ago (Mya) and Ad-β ~ 50–57 Mya (Wang et al. 2018), but these were different from some previous reports in which the age estimate for Ad-α was about 28 Mya and for Ad-β about 64–87 Mya (Xia et al. 2017) after the divergence of kiwifruit and tea (~ 80 Mya) (Wei et al. 2018). The evolutionary history of kiwifruits as well as the earlier diverged asterid clades are thus needed for further investigation.

The current genomic resources of kiwifruit plants are mainly from Ac cultivars “Hongyang” and “Red5” (Huang et al. 2013; Pilkington et al. 2018; Wu et al. 2019). A chromosome-scale genome assembly of an Ae cultivar “White” was also released (Tang et al. 2019), but the genomic information in relation to trait evolution of Ae is needed to be further investigated. Here we reported a high-quality chromosome-scale genome assembly of Ae by using a sample which was directly collected from the wild populations, representing variable genotype resource for this important kiwifruit species. We compared it to the released Ae cultivar “White” and Ac “Hongyang” v3 genomes from Kiwifruit Genome Database (Yue et al. 2020) to obtain the common and species-specific genomic contents. We characterized the evolutionary pattern of WGD events and the possible mechanism triggering trait variations of Ae. We also combined transcriptome-derived SNP data to dissect genetic relationships between Ae and other diverse *Actinidia* taxa. Our results will facilitate breeding and germplasm innovation of world kiwifruit industry.

## Results

### A high-quality genome assembly

We selected a wild Ae plant for sequencing. The estimated genome size of this sample is of ~ 689 Mb based on a *k*-mer analysis (Supplementary Fig. 1) and ~ 750 Mb by using flow cytometry analysis. The level of genomic heterozygosity is 0.98% (Supplementary Fig. 1 and Supplementary Table 1). We generated 60.13 Gb PacBio single-molecule long reads (N50 = 14.7 kb), 79.54 Gb Illumina paired-end short reads (with library insert sizes of 350 Kb), and 105.30 Gb 10× Genomics linked reads (Supplementary Fig. 2 and Supplementary Table 2). We firstly performed a PacBio-only assembly with the additional step of phasing contigs. The resulting assembly was polished with both PacBio long reads and Illumina short reads. We next assembled contigs into scaffolds based on linked reads. The resulted contig sequences were 655 Mb with N50 of 2.00 Mb, and scaffold sequences are 657 Mb with N50 of 5.07 Mb (Supplementary Table 3).

**Fig. 1:**
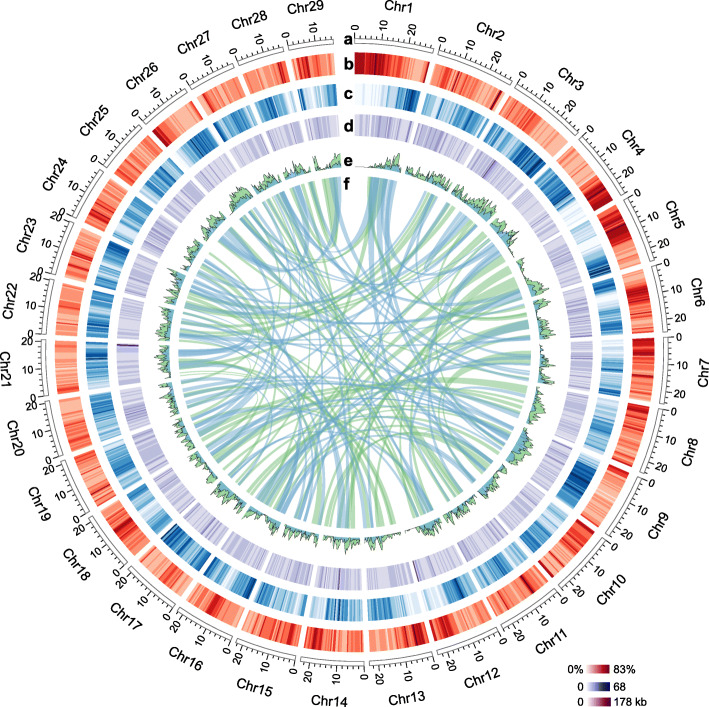
A high-quality genome assembly of *Actinidia eriantha* (Ae). **a** Circular representation of the chromosome-scale pseudomolecules. **b** The percentage of transposable-element coverage in sliding windows of 0.5 Mb. **c** Gene density in sliding windows of 0.5 Mb. **d** Length distribution of the presence/absence-variation (PAV) genes between Ae and *Actinidia chinensis* (Ac). **e** Genes evolved in relation to the respective Ad-α (light green) and Ad-β (light blue) whole-genome duplication (WGD) events. **f** The syntenic regions (only >N50 size shown here) involved into the Ad-α (light green) and Ad-β (light blue) WGDs, respectively

The assembly was further improved using high-throughput chromatin conformation capture (Hi-C) map, resulted into a final reference scaffold assembly comprised 29 unambiguous chromosome-scale pseudomolecules covering 95.85% (~ 629 Mb) of the genome assembly (hereafter referred to as chromosomes), in which the minimal length of chromosome was greater than 14.9 Mb (Fig. 1 a and Supplementary Fig. 3, Supplementary Table 4). The accuracy and completeness of the assembly were assessed by aligning Illumina short reads back to it, resulting into a mapping rate of 97.8%, with 97.1% of the assembly covered by at least three reads. Furthermore, > 97.7% of the de novo assembled transcripts could be mapped back to the assembly; and 236 out of 248 core eukaryotic genes mapping approach (CEGMA) genes are complete in the assembly. We also investigated benchmarking universal single-copy orthologs (BUSCO) in the assembly, as a result 93% of the BUSCOs were completely presented, and 1.5% were fragmentedly presented (Supplementary Table 5). All these collectively suggested that the quality of our genome assembly is high, comparable to the reported Ac v3 and Ae cultivar genomes (Tang et al. 2019; Wu et al. 2019), and also better than the other older versions of the Ac genomes (Supplementary Table 3).

### Genomic content and recent burst of LTR retrotransposons

Both homology-based and de novo approaches were used to investigate the repetitive DNA elements in the Ae genome. A total of 41.3% (271 Mb) of the Ae genome assembly was identified as repetitive sequences (Fig. 1 b, Supplementary Table 6), similar to those reported in the Ac (~ 36.0–43.4%) and Ae cultivar (43.3%) genomes. Long-term repeat (LTR) retrotransposons are predominant repetitive elements (196 Mb, 29.9% of the assembly), followed by about 40 Mb (6.1%) of DNA transposons, whereas the remainder was either assigned to other transposable elements (TEs) or tandem repeat families or could not be assigned (Supplementary Table 7). The composition of the different classes of repetitive DNA in Ae was also similar to that in the Ac v3 genome. We further identified 4005 and 3839 intact *Gypsy* and *Copia* retrotransposons, which belong to ten and seven *Gypsy* and *Copia* families, respectively (Supplementary Table 8). On the basis of these intact LTRs, the estimated bursts of both *Gypsy* and *Copia* retrotransposons occurred very recently (within one Mya; Supplementary Fig. 4).

We predicted a total of 41,521 high-confidence protein-coding genes from the Ae genome using an integrated strategy combining ab initio, transcript-based and homology-based predictions, with an average coding-sequence length of 1.1 kb and an average of 5.0 exons per gene (Fig. 1 c), which were close to those 40,464 genes annotated in the Ac v3.0 genome and 42,988 genes predicted in the Ae cultivar genome. Of these predicated genes, 78.3% (32,509) were expressed in vegetative and reproductive tissues and 99.4% (41,270) had substantial homology with known proteins or functional domains. Moreover, the gene elements in Ae, including lengths of mRNAs, distribution of CDS, and exons and introns, are comparable to those of Ac and five other representative plants, including tea (*Camellia sinensis*) in the Ericales, the sunflower (*Helianthus annuus*) as representative of asterids II, coffee (*Coffea canephora*) as a representative of asterids I, and grape (*Vitis vinifera*) (Supplementary Fig. 5). Two thousand eight hundred forty-five genes were identified as transcription factor (TF) genes, including 225 bHLH and 181 MYB genes (Supplementary Table 9). We also annotated noncoding RNA (ncRNA) genes, yielding 662 transfer RNA (tRNA) genes, 253 ribosomal RNA (rRNA) genes, 1411 small nuclear RNAs (snRNAs) and 1820 microRNA genes (miRNAs).

### Structural variations between ae and ac genomes

We aligned the Ae chromosomes to the Ac v3 genome and the Ae cultivar genome, respectively. Approximately 60.0% of the Ae genome have one-to-one syntenic blocks with 60.3% of the Ac genome sequence, while 83.3% of the Ae genome can be mapped to the 79.4% Ae cultivar genome in one-to-one syntenic pattern (Supplementary Fig. 6 and Supplementary Table 10). The nonsyntenic sequences were mostly DNA repeats, including transposable elements and dispersed genes. For the aligned one-to-one syntenic blocks between Ae and Ac genomes, we identified 15,628,085 single nucleotide polymorphisms (SNPs) and 3,766,293 small insertions and deletion polymorphisms (indels), with an average of 24 SNPs and six indels per kilobase (Supplementary Table 10). For the longer syntenic blocks between both Ae genomes, 8,181,896 SNPs and 6,617,383 indels were found, with an average of 12 SNPs and 10 indels per kilobase (Supplementary Table 10). Comparative analysis further revealed 23,409 Ae and 29,947 Ac genes with corresponding orthologous genes or gene fragments in their syntenic blocks, of which 13,942 Ae and Ac genes had no amino acid changes. Moreover, 31,499 genes in our Ae genome are orthologous ones or gene fragments of 24,361 genes in the Ae cultivar genome, of which 17,273 genes are conserved in their amino acid sequences.

By further comparison between the Ae and Ac v3 genomes, we identified 36,697 Ae specific genomic segments (~ 27.7 Mb) and 64,815 Ac specific genomic segments (~ 53.2 Mb) with length > 500 bp, which represented the presence/absence-variation (PAV) between them. Of which, 146 (in total 1,112,455 bp) and 401 (3,457,203 bp) PAV sequences in the respective Ae and Ac genomes were longer than 5 kb and that they were unevenly distributed across both genomes with some clusters (Fig. 1 d and Supplementary Fig. 7). We also identified 282 and 427 Ae- and Ac-specific PAV genes, respectively. Similarly, 10,854 Ae (~ 9.4 Mb) and 24,165 Ae cultivar (~ 19.8 Mb) specific genomic segments (> 500 bp) were identified, of which 77 (566,023 bp) and 165 (1,257,547 bp) PAV sequences (> 5 kb) in the respective Ae and Ae cultivar genomes were found, including 170 and 187 Ae and Ae cultivar specific genes. A total of 28,863 orthogroups were further identified among the three genomes, including 18,454 ones commonly presented in them, while 695 (1756 genes), 397 (873 genes) and 829 (2053 genes) specifically occurred in the Ac, Ae and Ae cultivar genomes, respectively.

Functional annotation demonstrated that some of the Ae-specific PAV genes were related to specific functions. For example, three PAV genes *scaf_25.6*, *scaf_75.500* and *scaf_75.501* in our Ae genome are significantly enriched in Gene ontology (GO) category of defense response to other organism (GO:0098542), and seven genes (*scaf_125.331*, *scaf_28.166*, *scaf_49.693*, *scaf_78.468*, *scaf_84.195*, *scaf_86.149*, *scaf_86.150*) are related to cell wall organization or biogenesis (GO:0071554) (*P* < 0.05). Twelve orthologous genes specific to the Ae genome were also found to be enriched in GO term of response to biotic stimulus (GO:0009607) (Supplementary Table 11). To trace the possible origin of these PAV genes, we aligned genome-wide resequencing reads of 10 kiwifruit backbone taxa on both the Ae and Ac genomes. These backbone taxa were previously identified by a phylogenomic analysis and they were thought to represent the core diversity present in the *Actinidia* genus (Liu et al. 2017). The majority of hits (~ 90.7% PAV genes) corresponded to homologs presented in at least one of the relatives of Ae or Ac (Supplementary Fig. 8), suggesting that the retention of ancestral polymorphism and/or extensive introgressions or hybridization occurred across diversified kiwifruit taxa in the wild.

### Genome evolution as an early diverging asterid lineage

**Fig. 2:**
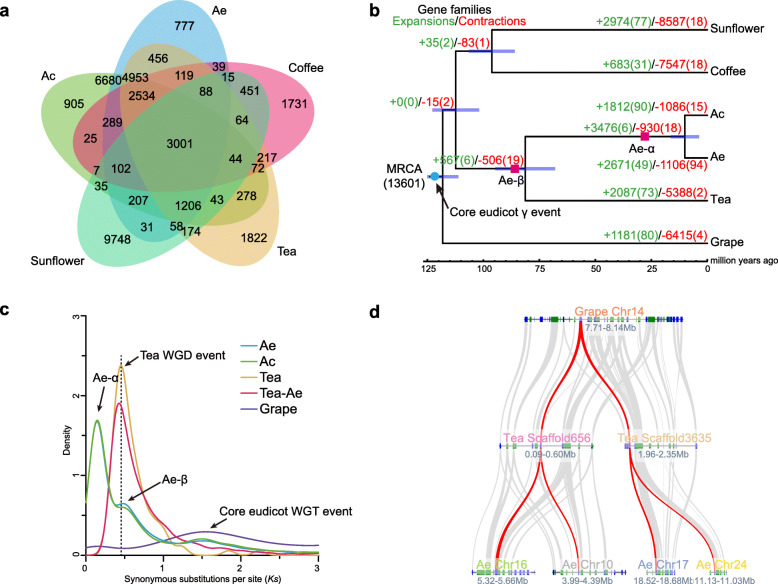
Evolution and comparative genomic analysis of *Actinidia eriantha* (Ae). **a** Number of gene families shared between Ae, *A. chinensis* (Ac) and three other asterid plants. **b** Phylogenomic analysis of Ae and other representative asterid plants. The potential whole genome duplication (WGD) events and expansion and contraction of gene families among these species are shown on the tree. The divergence time was estimated for each node. **c** Distribution of synonymous substitution rates (*Ks*) for pairs of syntenic paralogs in Ae and three other genomes. **d** A typical collinearity pattern between grape, tea and Ae genomes. Rectangles represent predicted gene models with color showing relative orientations (blue, same strand; green, opposite strand). Gray wedges connect matching gene pairs with one highlighted in red

We compared our Ae and the Ac v3 genomes with three other representative plants tea, sunflower and coffee in the asterid order, and also grape as an outgroup. Based on proteomes of these plants, we identified 23,204 orthologous gene families consisting of 167,770 genes (Supplementary Fig. 9). For clarity, we compare the gene families of the five asterid plants, of which a core set of 83,978 genes belong to 8094 gene families shared among these asterid species, whereas 919 genes from 497 gene families are unique to Ae (Fig. 2 a). Both Ae and Ac share the most gene families with tea, consistent with their close relationship within the Ericales (Fig. 2 b). With further analysis of gene family evolution, we found that 2671 gene families of Ae underwent expansion and 1106 gene families underwent contraction, of which 49 expanded (137 genes) and 94 contracted gene families (576 genes) were rapidly evolving (Fig. 2 b). Functional annotation demonstrated that the rapidly expanding gene families were enriched in gene ontology (GO) categories such as defense response to biotic stimulus (GO: 0009607) or response to freezing (GO: 0050826), suggesting the possible roles of these genes for the adaptation of Ae to harsh environments. We generated and dated a phylogenetic tree based on 1366 single-copy orthologous genes. The estimated divergence time of Ae and Ac was ~ 11 Mya, consistent with our recent estimation using resequencing data of multiple kiwifruit taxa (Liu et al. 2017). Moreover, the most recent common ancestor (MRCA) of Ae and Ac diverged from tea at about 81 Mya (Fig. 2b).

Based on the identified orthologous genes among Ae, Ac, tea and grape, as well as paralogous genes within each genome, we found that as expected, the established palaeohistory of Ae was consistent to those reported in Ac, in which three WGD events were identified, including the two kiwifruit-specific events (Ad-α and Ad-β) and the common core eudicot γ event (Fig. 2 b, c). Moreover, the Ad-β occurred before kiwifruit diverged from tea (Fig. 2c). The typical synteny pattern further reflected a 1:2:4 relationship between genomic regions from grape compared with both the tea and Ae genomes (Fig. 2d). We carried out a detailed characterization of the retention of duplicated genes during both kiwifruit-specific WGD events on the basis of pairwise synonymous substitution rates (*K*_s_ values) of paralogs. The *K*_s_ value in 0–0.335 corresponded to the Ad-α event and in 0.335–1 corresponded to the Ad-β event (Supplementary Fig. 10). We found that about 22,932 and 9415 genes are present in Ad-α and Ad-β events respectively (Fig. 1e, f), and they are significantly functionally enriched in 37 and 19 GO terms (corrected *p*-value < 0.05, Supplementary Table 12).

### Genes involved in ascorbic acid biosynthesis

**Fig. 3:**
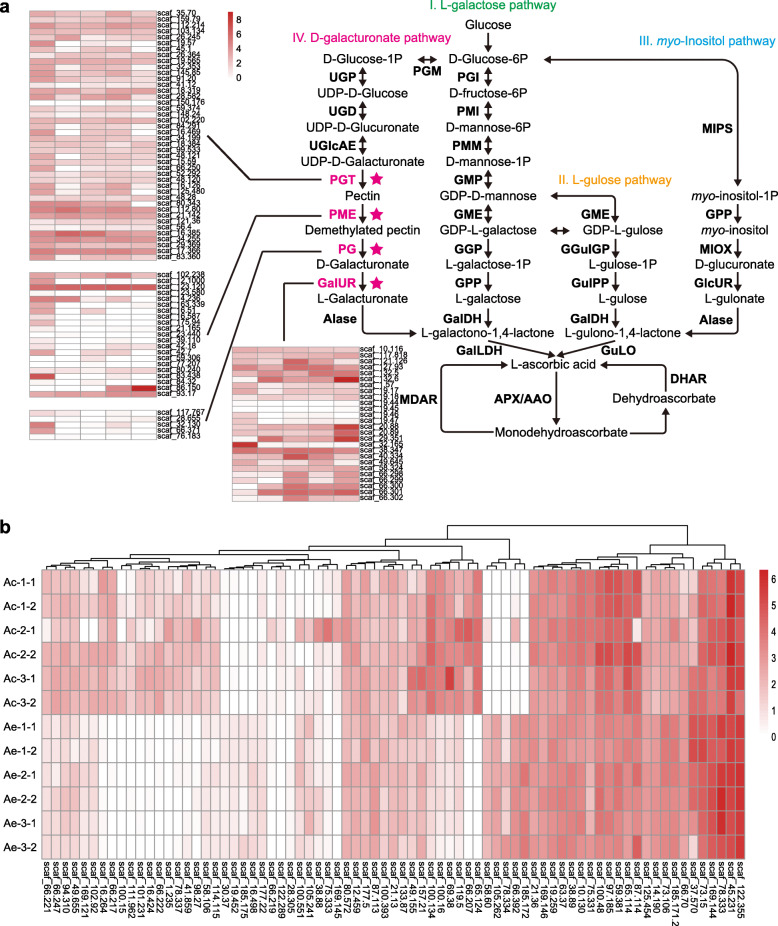
Evolution and expressions of genes in relation to ascorbic acid biosynthesis and disease resistance. **a** Evolutionary expansions of genes involved in the D-galacturonate pathway of ascorbic acid biosynthesis. Expressions of these genes in five different tissues (from left to right: fruit, flower, leaf, stem and root) were revealed. **b** Differential expressions of the common R genes between Ae and Ac. A previously available RNA-seq data was used and two sampling repeats in each of the three stages of Psa infection are shown

Kiwifruit is a rich dietary source of vitamin C and the content of vitamin C in Ae is three to four times than that of Ac (Huang 2014). We investigated and compared genes involved in the ascorbic acid biosynthesis and regeneration pathway (Bulley et al. 2009) in both kiwifruit taxa. Although expansion in genes of both plants from the L-galactose, L-glucose and Glucuronate/*myo*-Inositol biosynthesis pathways were not significantly different, we did find that gene families, including PGT (Polygalacturonate 4-α-galacturonosyltransferase), PME (Pectin methylesterase), PG (endopolygalacturonase), and GalUR (Galacturonic acid reductase) involved in the D-galacturonate pathway first described in strawberry (Agius et al. 2003; Rigano et al. 2018) were significantly expanded in Ae (Fig. 3 a; Supplementary Table 13). Most of these expanded genes were expressed, in particular for being expressed higher in fruits of Ae (Fig. 3 a), and some of them were randomly selected and further validated using quantitative real-time polymerase chain reaction analysis (Supplementary Fig. 11 and Supplementary Table 14). In the Ae genome, we further identified a significant increase of AMR1 (ascorbic acid mannose pathway regulator 1) copies which negatively regulates the L-galactose biosynthetic pathway in Arabidopsis (Zhang et al. 2009), and a decreased number of ERF98 (ethylene response factor subfamily b-3 of ERF/AP2 transcription factor family), a regulation gene contributing to ascorbic acid biosynthesis in Arabidopsis (Zhang et al. 2012). The high vitamin C accumulation in Ae fruit is thus possibly largely reinforced by the D-galacturonate pathway similar to that reported in strawberry and tomato (Rigano et al. 2018), although the contribution from the L-galactose pathway could be also equally important.

### Evolution and differential expression of disease-resistance genes of ae

We investigated those disease-resistance (R) genes in relation to both the pathogen-associated molecular pattern-triggered immunity (PTI) and the effector-triggered immunity (ETI) in the Ae genome. We identified 224 putative pattern-recognition receptor genes, which encode receptor-like kinases with a leucine-rich repeat domain (RLK-LRR, RLK for short) potentially contributed to PTI immunity. This number is somewhat less than the 263 RLK genes identified in Ac. We further found 95 nucleotide-binding site-LRR (NBS-LRR, NBS for short) genes which are possibly involved in the ETI immunity of kiwifruit. The number of NBS genes was consistently less than the 139 NBS genes found in the Ac genome. Many NBS genes found in both Ae and Ac genomes are truncated and the distribution of genes across different classes are distinct between two genomes (Supplementary Table 15).

To examine the expressions of 75 R gene commonly belonging to Ae and Ac, we used the available RNA-seq data that are derived from three stages of Psa infection on leaf tissues of both Ae and Ac, including the day 0 (without inoculation), day 2 and day 14 post inoculation (DPI) (Wang et al. 2017). We found that most of these R gene expressions were different between Ae and Ac (Fig. 3 b). In particular, we identified five genes that were especially expressed in Ae and six genes that were uniquely expressed in Ac, respectively (Supplementary Table 16). These genes are potentially important resistance genes in plants (Meyers et al. 2005) and possibly in relation to the distinct resistance/susceptibility between Ae and Ac against biotic and abiotic stress such the Psa invasions. We take a RPS2-like gene (*scaf_105.262*) especially expressed in Ae as an example. In *Arabidopsis*, RPS2 controls specific recognition of *P. syringae* strains expressing the avirulence gene avrRpt2 (Kunkel et al. 1993). A similar ETI-layer mechanism was therefore possible to confer resistance of Ae on Psa.

### The genetic relationship between ae and other kiwifruit taxa

Diverse kiwifruit taxa can be distinguished by their fruit skin types, such as with soft and hairless skins (SHS), rough and warty skins (RWS) or rough and hairy skins (RHS). Ae is a member of the RHS group, and it has particularly milky white or sometimes pale brown fruit hairs, which are clearly distinct from those of many other kiwifruit taxa (Huang 2014). To assess genetic relationship between Ae and other kiwifruit taxa, we performed transcriptome sequencing of 21 kiwifruit samples belonging to 15 representative *Actinidia* taxa (Supplementary Table 17). We generated ~ 150 Gb RNA-seq reads with an average of 50 million reads per sample. Mapping of these RNA-seq reads to the Ae reference genome identified 3,414,917 SNPs and 328,013 small indels.

**Fig. 4:**
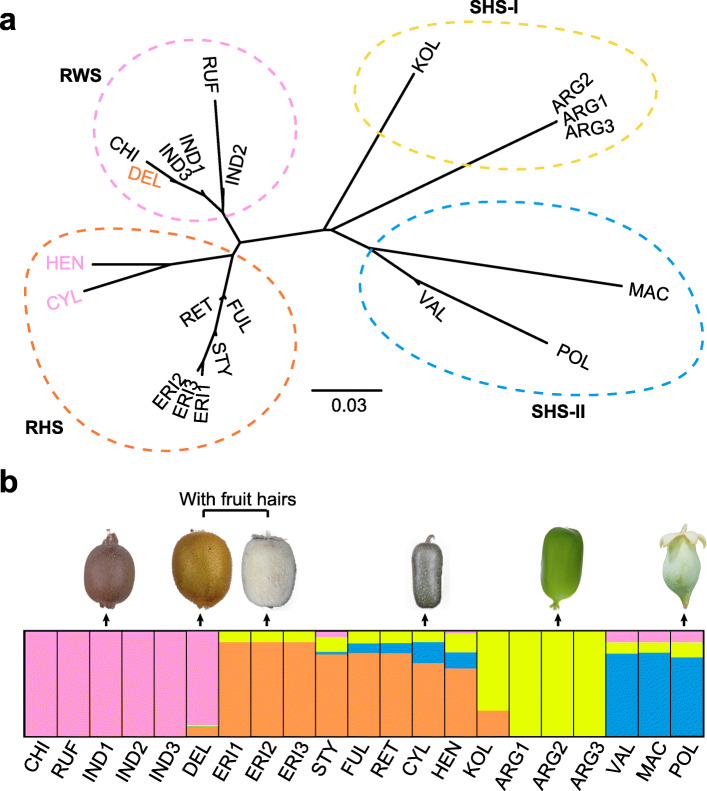
Evolutionary relationship of *Actinidia* taxa with diverse fruit skin types. **a** A neighbor-joining tree showing the genetic relationship of species investigated. Two subgroups I and II of SHS are identified. **b** Results of STRUCTURE analyses revealing widely genomic admixture of many *Actinidia* taxa. See Supplementary Table 17 for the full name of the abbreviation of each taxon. RHS: rough and hairy skins; RWS: rough and warty skins; SHS: soft and hairless skins

We examined genetic admixture among these samples using both Neighbor-joining (NJ) tree and STRUCTURE analyses based on transcriptomic data-derived SNPs. Generally, samples were clustered according to the three defined fruit skin types (Supplementary Table 17), with a further subdivision of the SHS group into two subgroups (Fig. 4a). However, both RWS taxa *A. cylindrica* (CYL) and *A. callosa* var. *henryi* (HEN) were clustered with those of RHS samples despite their rough and warty fruit skins (Fig. 4a). Conversely, the RHS taxon *A. chinensis* var. *deliciosa* (DEL) grouped with RWS samples. With the best estimate of *K* value of 4 for the STRUCTURE analysis (Supplementary Fig. 12), we found a similar relationship between kiwifruit samples investigated (Fig. 4b). Notably, we can identify widespread genomic admixture of many kiwifruit taxa which presented phylogenetic-phenotypical discordance, including CYL, HEN and DEL mentioned above. For DEL and ERI (Ae), both of which have fruit hairs, their genomic compositions are distinctly different. The genomic contents of DEL, a variety of *A. chinensis* (CHI), are mainly similar to those of CHI, despite possibly partial introgression from those of Ae. Comparatively, the observed genomic components of Ae possessed introgressive contents from those of the SHS group (Fig. 4b).

## Discussion

Here we presented a high-quality genome assembly of Ae, an economically important wild relative of the cultivated kiwifruits. Our assembly was greatly improved from that of older versions of kiwifruit genomes (Supplementary Table 3) (Huang et al. 2013; Pilkington et al. 2018) and also comparable to the recently reported versions (Tang et al. 2019; Wu et al. 2019), in particular for the repetitive contents in which more intact repetitive elements were identified and classified (Supplementary Table 8). We found very recent presence of these intact repetitive elements in the Ae genome (Supplementary Fig. 4). The recent LTR retrotransposon bursts are thought to be an important factor contributing to high level of genomic repetitive contents and large genome size in plants such as those reported in both chrysanthemum (Song et al. 2018) and wheat genomes (Ling et al. 2018). Comparatively, the presence of the recent burst of LTRs in the Ae genome is relatively weak, consistent with its moderate level of genomic repetitive contents (41.3% of the genome assembly) and the relatively compact genome size (~ 689 Mb).

The genome of Ac was shown to have undergone two WGD events (Wang et al. 2018). Our analysis confirmed these events and also demonstrated their presense in the evolution of Ae genome (Fig. 2b and c). However, it is unclear if the kiwifruit Ad-β event was shared with tea, a closely related asterid plant, although a previous paper provides weak evidence (~ 16% gene trees) supporting the Ad-β event occurred prior to the divergence between tea and kiwifruit (Xia et al. 2017). With improved assembly of both kiwifruit (Ae) and tea genomes (Xia et al. 2017; Wei et al. 2018), we provided confirmed evidence that a shared WGD event (Ad-β) occurred before the divergence of kiwifruit and tea (Fig. 2c). Moreover, because the evolutionary divergence between kiwifruit and tea occurred at ~ 81 Mya (Fig. 2b) as that also reported in a recent study (Wei et al. 2018), the age estimate for Ad-β event is thus possibly > 80 Mya. This is consistent with two previous estimates of about 64–87 Mya (Xia et al. 2017) but distinctly different from a recent reanalysis of results on the basis of the Ac genome data, which suggested a range of ~ 50–57 Mya for the presence of the Ad-β event (Wang et al. 2018).

To date, the involvement of the D-galacturonate pathway in controlling the ascorbic acid pool of kiwifruit remains unclear, given the L-galactose pathway is considered the main pathway in plant tissues (Wheeler et al. 2015) as thus also in kiwifruit. In cultivated kiwifruit, the ascorbic acid pool generally increased over time, peaking at breaker stage and then decreasing (Li et al. 2010). This pattern is similar to that observed in tomato. A recent study showed that ascorbic acid biosynthesis from the D-galacturonate pathway contributed to the high ascorbic acid content in an introgressed line of tomato (Rigano et al. 2018). A similar pattern was possibly present in Ae, in which the expanded and numerous expressed genes of the D-galacturonate pathway in Ae may contribute to its relatively high ascorbic acid contents. We found a reduced number and differential expressions of disease-resistance genes of Ae versus those of Ac. The plant immune system is complex, including both layers of PTI and ETI (Jones and Dangl 2006). As a main epidemic of the cultivated kiwifruit domesticated from Ac, the evolution of Psa may potentially be more specific to its host Ac, leaving Ae free from the canker burst. Our analysis provided clues for possible ETI-related immunity of Ae in the face of Psa invasions, such as the RPS2-like genes. Further analyses are needed to dissect the mechanism underlying kiwifruit resistance/susceptibility to Psa.

The evolutionary relationship of kiwifruit plants is relatively complex, and interspecific hybridization or introgressive gene flow have significantly contributed to their morphological diversification (Liu et al. 2017). The diverse fruit skin types (with or without hairs) are the most predominant characteristic to distinguish different kiwifruit species and varieties. The evolutionary origin of kiwifruit trichomes such as those present on the *A. chinensis* var. *deliciosa* and Ae fruit surface is potentially polyphyletic (Fig. 4b). A previous study of electron microscopy showed that the fruit surface of *A. chinensis* var. *deliciosa* has only small, uniseriate hairs, whereas those in Ae have both small, uniseriate, two-celled hairs with a long tapering apical cell and abundant, much longer, slender, multicelled hairs, often branching near the base to form a cluster of three hairs, each terminating in a very long thin apical cell (White 1986). The trichome type on the fruit surface of *A. chinensis* var. *deliciosa* and Ae are thus clearly different, consistent to their different pattern of hybridization contributing to alternative genomic components (Fig. 4b).

Introgressing traits from wild relatives for germplasm improvements and innovations is particularly important in breeding of both crops and horticultural plants. Our analysis of the genome and evolution of a wild kiwifruit Ae therefore represents a key step in introgressive breeding of kiwifruit. The genome structure of Ae, including the variation of PAV genes, was distinct from that of Ac. All these collectively provided clues in terms of the molecular basis of trait diversification among different kiwifruit taxa. These are highly valued for breeding applications. Currently, kiwifruit cultivars derived from hybrids between Ae and Ac have already showed their success in terms of fruit quality and disease resistance in world kiwifruit industry. With improved genomic resources including the Ae genome presented here, future kiwifruit introgression breeding can combine more desirable traits from its wild relatives.

## Methods

### Sample collection

The *A. eriantha* (Ae) and other *Actinidia* taxa samples used for both genomic and transcriptomic analyses in the present study were obtained from the National *Actinidia* Germplasm Repository (NAGR; Wuhan, China). Compared to the Ae cultivar “White”, the Ae plant used here was originally derived from the wild populations of Hunan province without further artificial domestication, representing potentially genetic variation in relation to nature selection and adaptation. Young leaves of Ae were collected for genomic sequencing, and tissue materials from root, stem, flower and fruit were used for RNA-seq and genomic annotation. We collected fruit peels from 21 kiwifruit samples belonging 15 *Actinidia* taxa for comparative transcriptomic analyses. Due to the relatively widely natural distribution of the three species Ae, *A. arguta*, and *A. indochinensis*, we included three different samples for each of these species in our analyses (Supplementary Table 17).

### DNA extractions, library preparation and sequencing

We extracted total genomic DNA from young leaves using the traditional cetyltriethylammnonium bromide (CTAB) method with minor modifications. The DNA quality was monitored on 1% agarose gels, purity was checked with a NanoPhotometer spectrophotometer (Implen, Munich, Germany) and the concentration was measured using the Qubit 2.0 Fluorimeter (Life Technologies, Carlsbad, CA, USA).

To obtain long reads, we performed whole-genome shotgun sequencing using SMRT sequencing technology (Pacific Biosciences, California, USA). DNA libraries with large inserts were prepared with the PacBio template preparation kit (DNA Template Prep Kit 1.0) and sequenced on the PacBio Sequel platform. Subread filtering was performed by using PacBio SMRT analysis software (v2.3.1) with the parameter of minimum length of 50. A paired-end library with 350 bp insert size was further constructed and sequenced on the Illumina HiSeqX Ten sequencing platform (Illumina, San Diego, CA, USA). 10× Genomics libraries were constructed following the manufacturer’s protocol (10× Genomics), and were sequenced into standard Illumina paired-end reads as linked-reads.

### Estimation of genome size and heterozygosity

We estimated the genome size of Ae using a *k*-mer (*k* = 17) analysis based on Illumina paired-end short reads. The software Jellyfish (v2.1.4) (Marçais and Kingsford 2011) was used for counting of *k*-mers in DNA. GCE (v1.0) (Liu et al. 2013) was used for estimating the genome size. A further investigation was conducted based on flow cytometry. The 2C DNA content of Ae was determined on a BD Accuri™ C6 flow cytometer (BD Biosciences, San Jose, CA, USA) and compared with that of Ac “Hongyang”. We investigated the level of genomic heterozygosity based on the percentage of heterozygous *k*-mer species.

### Hi-C library preparation and sequencing

Hi-C libraries were constructed from fresh leaves of Ae as described previously (Xie et al. 2015). Briefly, leaf tissues were fixed in formaldehyde and lysed, and then the nuclei were extracted. Chromatin with cross-linked DNA was digested with DpnII restriction enzyme (Takara) overnight. Sticky ends were labeled by biotin and ligation between cross-linked fragments was performed to form chimeric junctions that were enriched for. The resulting DNA was sheared to a size of 500–700 bp and DNA ends were subsequently repaired. The Hi-C libraries were sequenced on the Illumina HiSeq 2500 platform to obtain 150-bp paired-end reads. After filtering, a total of 937,308,228 clean paired-end reads were obtained.

### Scaffolding and chromosome-scale genome assembly

We used both high-depth PacBio long reads and 10× Genomics linked reads to assemble high-quality contigs and contiguous scaffolding. Falcon (Chin et al. 2016) was firstly used to correct the PacBio long reads and assemble them into contiguous sequences with parameters: length_cut-off_pr = 4000, max_diff = 100, max_cov = 100. This resulted in primary contigs which were then polished using Quiver (Chin et al. 2013) by aligning PacBio reads, and using BWA (Li and Durbin 2009) by aligning Illumina paired-end short reads. The Pilon (v1.22) (Walker et al. 2014) program was run with default parameters to fix bases, fill gaps, and correct local misassemblies. The error-corrected assembly were then scaffolded by the FragScaff software (v 1.1) (Adey et al. 2014) using the 10× barcoded reads. The alignment of Hi-C reads on the Ae genome was finally conducted and was statistically analyzed to recognize effective interactions. Agglomerative hierarchical clustering and LACHESIS (Burton et al. 2013) were used to cluster scaffolds into chromosome-scale assembly map with a karyotype of 2*n* = 58.

### Genome quality assessment

To investigate possible sequence contamination, we examined the distribution of GC contents in each of the non-overlapping 10-kb windows. This resulted into a clear distribution peak at around 35.65% without further discreteness, suggesting a lack of foreign sequence contamination. To evaluate the correctness of the assembled Ae genome, we mapped Illumina short reads and de novo assembled transcripts back to the genome assembly using BWA. To examine the completeness of the assembly, Core Eukaryotic Genes Mapping Approach (CEGMA) (Parra et al. 2007) and Benchmarking Universal Single Copy Orthologs (BUSCO, v2.0) (Simão et al. 2015) analysis were further conducted.

### Genome annotation

We identified repetitive sequences in the Ae genome using the combined tools in RepeatModeler (v1.0.4) (https://github.com/rmhubley/RepeatModeler), RepeatMasker (v4.0.5) (http://www.repeatmasker.org/), PILER (Edgar and Myers 2005) and LTR_finder (Xu and Wang 2007). The integration times (*t*) of intact LTRs were estimated with the equation *t* = *K*/*2r*, where *K* is the number of nucleotide substitutions per site between each LTR pair and *r* is the nucleotide substitution rate, which was set to 1 × 10^− 8^ substitutions per site per year (Strasburg and Rieseberg 2008).

Putative protein-coding genes in the Ae genome were predicted by using the Maker package (v2.31.8) with protein references from published plant genomes and the transcriptome data generated in this study. We also used AUGUSTUS (v3.2.2) (Stanke et al. 2006) to de novo predict gene structures. The rRNAs and tRNAs were predicted with RNAmmer (v1.2) (Lagesen et al. 2007) and tRNAscan-SE (v1.23) (Lowe and Eddy 1997), respectively. Other ncRNAs were identified by using the Perl program Rfam_scan.pl with inner calling conducted with Infernal (v1.1.1) (Nawrocki and Eddy 2013). Functional annotation of protein-coding genes was performed with BlastP (e-value cut-off 1e–05) and searches for gene motifs and domains were conducted with interProScan (v5.28) (Jones et al. 2014).

We obtained the GO terms of genes from the corresponding InterPro or Pfam entry and we used KOBAS (v2.0) (Xie et al. 2011) and the KEGG database to reconstruct related pathways. All functional enrichment analyses of significant genes in the present study were performed by using clusterProfiler package (v3.16) of R program (v4.0.2) (Yu et al. 2012). The enriched functional terms with *P* value < 0.05 were considered as significant.

### Identification of SNPs, indels and PAV variation between ae and ac genomes

The SNPs and indels (< 100 bp) between the Ae and Ac (Hongyang v3) or Ae cultivar (White) genomes were identified with Mummer (v4.0.0beta2) (Marçais et al. 2018) based on their one-to-one syntenic blocks. The corresponding pseudochromosomes in a comparison were mapped with NUCmer. The delta-filter was used to remove mapping noise and confirm the alignment blocks one by one. Finally, show-snps was used to obtain SNPs and indels (< 100 bp).

We identified PAV sequences between the Ae and Ac genomes through a sliding-window method (Sun et al. 2018). To identify Ae specific sequences, we divided the Ae genome into 500-bp overlapping windows with a step size of 100 bp and then aligned each 500-bp window against the Ae and Ac genomes with BWA. The sequences of windows that could not be aligned, or that aligned to the Ac genome with a primary alignment coverage < 25% but that could be properly aligned to the Ae genome, were defined as Ae-specific sequences. Overlapping windows that could not be aligned were merged. We identified Ac specific sequences through the same method.

We merged the CDS of different transcripts to represent a single gene, and we defined a PAV gene if > 75% of the CDS region was covered by corresponding PAV sequences. We further aligned Ae/Ac resequencing reads derived from the our recent phylogenomic project (Liu et al. 2017) to the Ae/Ac genome with BWA mem to exclude potential false positives. For the Ae/Ac-specific genes, we filtered those with > 50% CDS regions covered by Ae/Ac reads to obtain the final PAV genes. Resequencing reads of ten kiwifruit taxa, including *A. latifolia*, *A. lanceolata*, *A. cylindrica*, *A. fulvicoma* var. *hirsuta*, *A. collosa* var. *henryi*, *A. rufa*, *A. chinensis* var. *setosa*, *A. polygama*, *A. melanandra*, *A. hypoleuca* were also aligned to the Ae and Ac genomes to investigate the origin and evolution of these PAV genes.

### Gene family and phylogenomic analysis

Orthologous gene clusters between Ae and other representative plants including Ac, tea, sunflower, coffee and grape were identified using the OrthoMCL program (Li et al. 2003). Orthologs were classified as follows: single-copy orthologs are genes wherein no other paralogs are present in a family; multiple-copy orthologs are genes wherein one family contains at least two genes of this species and at the same time contains all other species; unique paralogs are genes wherein one family only contains genes of this species; and other orthologs are all other genes as well as non-clustered genes. We determined gene family expansion or contraction using CAFÉ (v3.0) (Bie et al. 2006).

We converted alignments from MUSCLE into coding sequences and used RAxML (v8.2.10) (Stamatakis 2014) to construct the phylogenetic trees. The Bayesian Relaxed Molecular Clock method was used to estimate species divergence times with the program MCMCTREE (v4.0) within the PAML package (v4.8) (Yang 2007). Published divergence times for Sunflower-Coffee (< 107 Mya, > 93 Mya), Kiwifruit-Tea (< 108 Mya, > 70 Mya), Sunflower-Tea (< 116 Mya, > 93 Mya) and Sunflower-Grape (< 124 Mya, > 110 Mya) were used to calibrate the divergence time. We used PAML to calculate the value under evolutionary pressure on the basis of 1366 single-copy gene families.

### Investigation of whole-genome duplication

We detected and compared WGD events between the Ae and three other plant genomes (Ac, tea and grape). Paralogous gene pairs were identified with Blast-based methods and syntenic paralogs were determined with MCScanX (Wang et al. 2012). We also identified orthologs between these plant genomes. We calculated the number of synonymous substitutions per synonymous site (*K*_s_) for gene pairs based on the NG method of Yang implemented in the PAML program (v4.8). The synonymous substitution rate of 8.25 × 10^− 9^ mutations per site per year for asterids was applied to estimate the ages of the WGDs (Badouin et al. 2017). To investigate the retention of duplicated genes from each of the kiwifruit-specific WGDs, we extracted all genes in the syntenic blocks and split them into two groups. The *K*_s_ value of syntenic blocks in 0–0.335 corresponded to Ad-α gene duplication and in 0.335–1 corresponded to the Ad-β event.

### Identification of genes related to ascorbic acid biosynthesis and disease resistance

To investigate genes in relation to ascorbic acid biosynthesis, we first searched the homologous genes in Ae on the basis of those genes previously identified in Ac (Bulley et al. 2009; Huang et al. 2013) using BlastP (e-value cut-off 1e-10). Further searching based on genes from model plant *Arabidopsis* as queries for homologs and conserved domains were also conducted. To identify disease resistance related genes, we retrieved and aligned protein sequences of both Ae and Ac using BlastP and these sequences were searched for conserved domains (RLK: PF00560; LRR: PF00069; NB-ARC: PF00931) using HMMER (v3.1b2) (http://hmmer.org/). We used both PfamScan (https://www.ebi.ac.uk/Tools/pfa/pfamscan/) and Paircoil2 (McDonnell et al. 2006) for further checking of other domains in relationto TIR, RPW8, CC and LRR in genes obtained. The common R genes between Ae and Ac was searched with BlastP (e-value cut-off 1e-10, max_target_seqs 1).

### RNA library preparation and RNA-seq data analysis of *Actinidia* taxa

RNA was extracted with the PureLink RNA mini kit (Life Technologies, Carlsbad, CA, USA). We constructed 26 RNA-seq libraries were constructed (including those derived from five plant tissues of fruit, flower, leaf, stem and root used for genome annotation and gene expression analyses, and those derived from fruit peels of 21 samples within 15 different *Actinidia* taxa). All these libraries were prepared with the Illumina TruSeq RNA sample preparation kit in accordance with the manufacturer’s instructions (Illumina, San Diego, CA, USA), and sequenced with the Illumina HiSeq 2500 platform. Raw RNA-seq reads were processed with Trimmomatic (v0.33) (Bolger et al. 2014) to remove adaptor and low quality sequences. Reads > 40 bp were kept and the ribosomal RNA database (https://www.arb-silva.de/) were used to filter reads. For gene expression analysis, the resulting clean reads were aligned to the assembled Ae genome using TopHat (Trapnell et al. 2009) with two mismatches allowed. Counts for each gene were derived and normalized to FPKM following alignments. To identify SNPs and small indels between Ae and the other *Actinidia* taxa, we mapped the resulting clean RNA-seq reads to the Ae genome with STAR (v2.6.1d) (https://github.com/alexdobin/STAR). Only reads uniquely mapped were kept and SNPs and indels were called by GATK (v3.8) (McKenna et al. 2010).

### Analysis of genetic relationship

To investigate the genetic relationship between Ae and other representative *Actinidia* taxa, we conducted both phylogenetic and genetic structure analysis of samples based on the transcriptomic data-derived SNPs (Supplementary Table 17). Only SNPs with minor allele frequency (MAF) > 5% and missing data < 20% (a total of 365,344) were used for both analyses. We constructed a maximum likelihood tree using RAxML. A subset of 9875 SNPs at 1/37 sites was used to construct genetic structure analysis using STRUCTURE (v2.3.4) (Pritchard et al. 2000).

### Supplementary Information

**Additional file 1: Supplementary Fig. 1.** 17-mer distribution analysis for genome size and heterozygosity estimation. **Supplementary Fig. 2.** Subreads length distribution of PacBio clean sequence data. **Supplementary Fig. 3.** Hi-C interaction and assembly of chromosome-scale pseudomolecules. **Supplementary Fig. 4.** Insertion burst of *Gypsy* and *Copia* retrotransposons in the *Actinidia eriantha* genome. **Supplementary Fig. 5.** The distribution of gene elements within six plant species. Ae: *Actinidia eriantha*, Ac: *A. chinensis*. **Supplementary Fig. 6.** The synteny between the *Actinidia eriantha* (Ae) and *A. chinensis* (Ac) pseudo-chromosomes. **Supplementary Fig. 7.** The length distribution of presence/absence-variation (PAV) sequences in *Actinidia eriantha* (Ae) and *A. chinensis* (Ac). **Supplementary Fig. 8.** Homologs of the *Actinidia eriantha*/*A. chinensis* presence/absence-variation genes across different *Actinidia* taxa. A red color indicates the presence of a gene while a blue color indicates the absence of a gene in a corresponding species or variety. **Supplementary Fig. 9.** Statistical analysis of orthologs or unique paralogs present in six plant species. **Supplementary Fig. 10.** Dividing Ae WGD events based on pairwise synonymous substitution rates (*K*_s_ values) of paralogs. **Supplementary Fig. 11.** The consistent gene expressions in both transcriptome data and quantitative real-time polymerase chain reactions. **Supplementary Fig. 12.** The best *K* value estimated for STRUCTURE analysis of diverse kiwifruit taxa. **Supplementary Table 1.** Genome survey summary based on a *k*-mer analysis. **Supplementary Table 2.** Summary of the sequencing data of *Actinidia eriantha*. **Supplementary Table 3.** Comparison of kiwifruit genome assembly statistics. **Supplementary Table 4.** Summary of scaffolds in each chromosome-scale pseudomolecules. **Supplementary Table 5.** Assessing *Actinidia eriantha* genome and annotation completeness with BUSCO analysis. **Supplementary Table 6.** Statistics of genomic repetitive contents of *Actinidia eriantha*. **Supplementary Table 7.** Classification of the transposable elements (TEs) in the *Actinidia eriantha* genome. **Supplementary Table 8.** Summary of both the intact *Gypsy* and *Copia* LTR families. **Supplementary Table 9.** The identified transcription factor (TF) genes in the *Actinidia eriantha* genome. **Supplementary Table 10.** Summary of aligned sequences, SNPs and Indels between Ae and Ac genomes. **Supplementary Table 11.** Enriched GO terms of the genes specific presented in our Ae genome. **Supplementary Table 12.** Enriched GO terms of duplicated genes specific presented in both Ad-α and Ad-β events respectively. **Supplementary Table 13.** Ascorbate-related genes investigated in both the *Actinidia eriantha* (Ae) and *A. chinensis* (Ac) genomes. **Supplementary Table 14.** Primers used for quantitative real-time polymerase chain reaction analysis. **Supplementary Table 15.** Nucleotide-binding site (NBS) genes identified in both *Actinidia eriantha* (Ae) and *A. chinensis* (Ac) genomes. **Supplementary Table 16.** Disease resistance genes specially expressed in *Actinidia eriantha* (Ae) and *A. chinensis* (Ac) respectively. **Supplementary Table 17.** List of *Actinidia* taxa used for transcriptomic sequencing.

## Data Availability

All data generated or analyzed during this study are included in this published article.

## Abbreviations

Ac: *Actinidia chinensis*
Ae: *A. eriantha*
PAV: presence/absence-variation
WGD: whole genome duplication
FAO: Food and Agriculture Organization
Psa: *Pseudomonas syringae* pv. *actinidiae*
Mya: million years ago
Hi-C: high-throughput chromatin conformation capture
CEGMA: core eukaryotic genes mapping approach
BUSCO: benchmarking universal single-copy orthologs
LTR: long-term repeat
TE: transposable elements
TF: transcription factor
GO: Gene ontology
MRCA: the most recent common ancestor
PGT: Polygalacturonate 4-α-galacturonosyltransferase
PME: Pectin methylesterase
PG: endopolygalacturonase
GalUR: Galacturonic acid reductase
AMR1: ascorbic acid mannose pathway regulator 1
ERF: ethylene response factor
PTI: pattern-triggered immunity
ETI: effector-triggered immunity
RLK: receptor-like kinase
LRR: leucine-rich repeat
NBS: nucleotide-binding site
DPI: day post inoculation
SHS: hairless skins
RWS: warty skins
RHS: rough and hairy skins
NJ: Neighbor-joining
CYL: *A. cylindrica*
HEN: *A. callosa* var. *henryi*
DEL: *A. chinensis* var. *deliciosa*
NAGR: the National *Actinidia* Germplasm Repository
MAF: minor allele frequency
