# Supplementary material for: The genome sequencing and comparative analysis of a wild kiwifruit Actinidia eriantha
Source: Mol Hortic. 2022 May 8;2:13. doi: 10.1186/s43897-022-00034-z (PMC10515239; doi:10.1186/s43897-022-00034-z)
Supplement: Supplementary file 1 — Additional file 1: Supplementary Fig. 1. 17-mer distribution analysis for genome size and heterozygosity estimation. Supplementary Fig. 2. Subreads length distribution of PacBio clean sequence data. Supplementary Fig. 3. Hi-C interaction and assembly of chromosome-scale pseudomolecules. Supplementary Fig. 4. Insertion burst of Gypsy and Copia retrotransposons in the Actinidia eriantha genome. Supplementary Fig. 5. The distribution of gene elements within six plant species. Ae: Actinidia eriantha, Ac: A. chinensis. Supplementary Fig. 6. The synteny between the Actinidia eriantha (Ae) and A. chinensis (Ac) pseudo-chromosomes. Supplementary Fig. 7. The length distribution of presence/absence-variation (PAV) sequences in Actinidia eriantha (Ae) and A. chinensis (Ac). Supplementary Fig. 8. Homologs of the Actinidia eriantha/A. chinensis presence/absence-variation genes across different Actinidia taxa. A red color indicates the presence of a gene while a blue color indicates the absence of a gene in a corresponding species or variety. Supplementary Fig. 9. Statistical analysis of orthologs or unique paralogs present in six plant species. Supplementary Fig. 10. Dividing Ae WGD events based on pairwise synonymous substitution rates (Ks values) of paralogs. Supplementary Fig. 11. The consistent gene expressions in both transcriptome data and quantitative real-time polymerase chain reactions. Supplementary Fig. 12. The best K value estimated for STRUCTURE analysis of diverse kiwifruit taxa. Supplementary Table 1. Genome survey summary based on a k-mer analysis. Supplementary Table 2. Summary of the sequencing data of Actinidia eriantha. Supplementary Table 3. Comparison of kiwifruit genome assembly statistics. Supplementary Table 4. Summary of scaffolds in each chromosome-scale pseudomolecules. Supplementary Table 5. Assessing Actinidia eriantha genome and annotation completeness with BUSCO analysis. Supplementary Table 6. Statistics of genomic repetitive contents of Actinidi [file 43897_2022_34_MOESM1_ESM.zip › Supplementary_information.docx]

**The genome sequencing and comparative analysis of a wild kiwifruit *Actinidia eriantha***

# Supplementary Figures

**
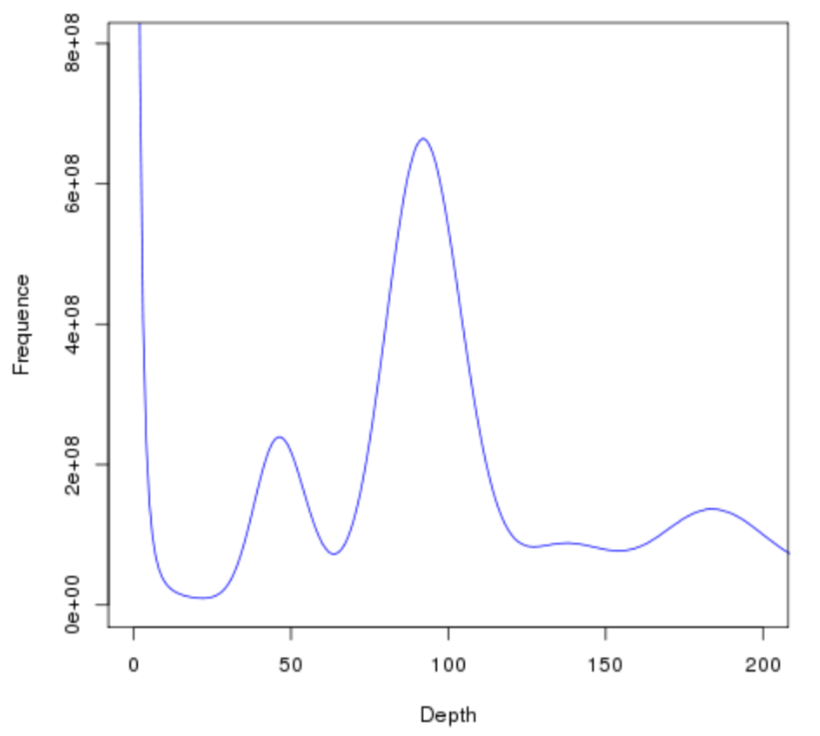
**

### Supplementary Fig. 1 17-mer distribution analysis for genome size and heterozygosity estimation.

**
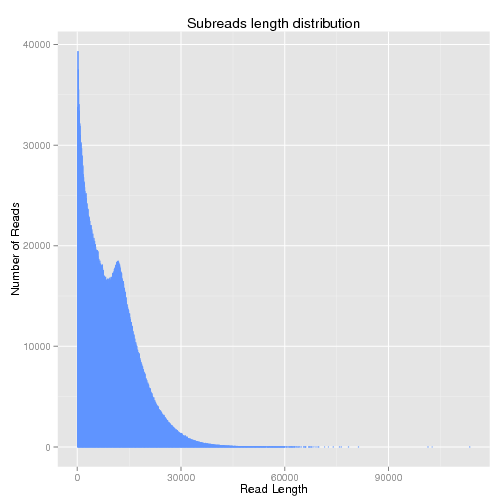
**

### Supplementary Fig. 2. Subreads length distribution of PacBio clean sequence data


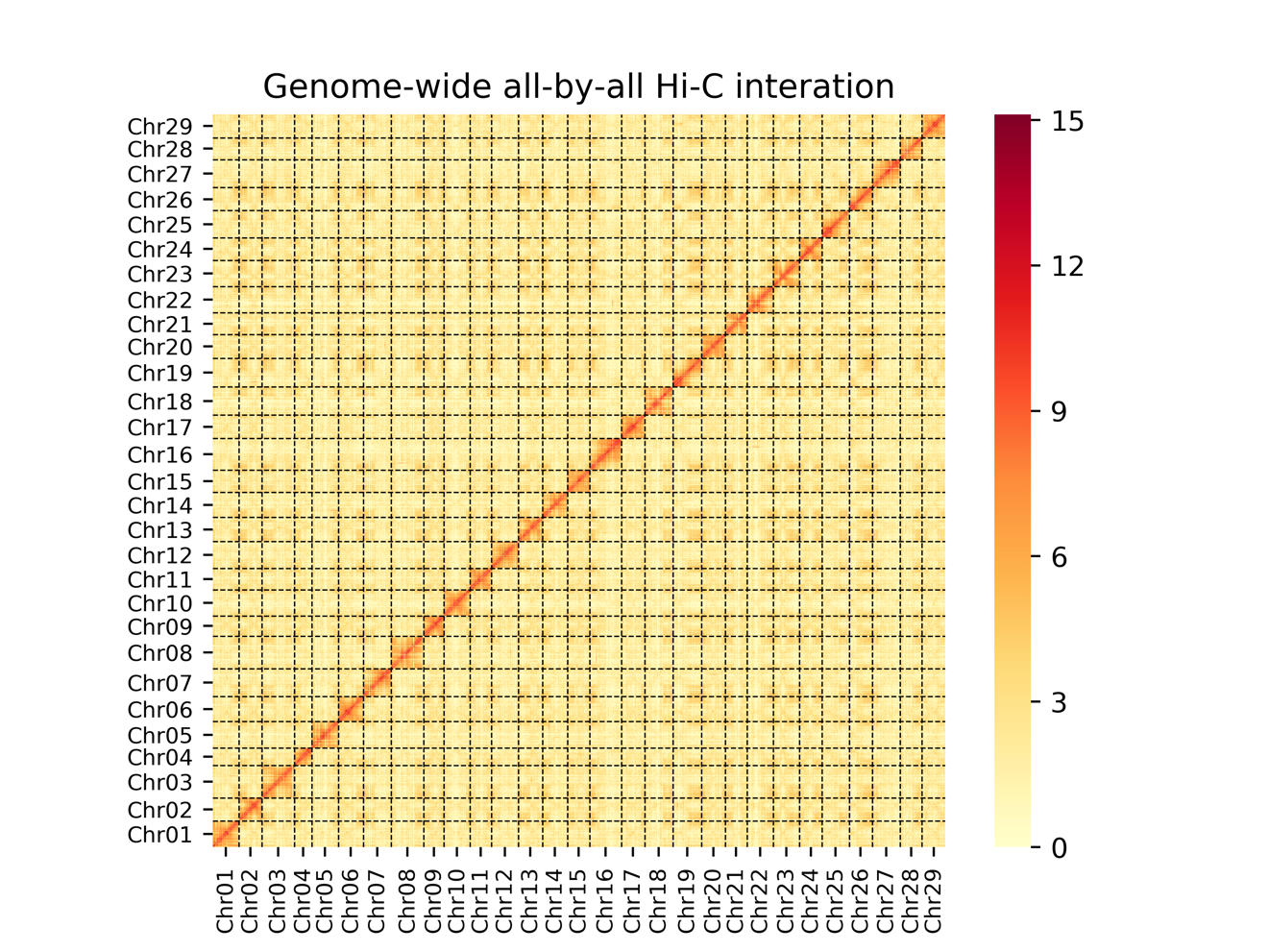


### Supplementary Fig. 3. Hi-C interaction and assembly of chromosome-scale pseudomolecules.


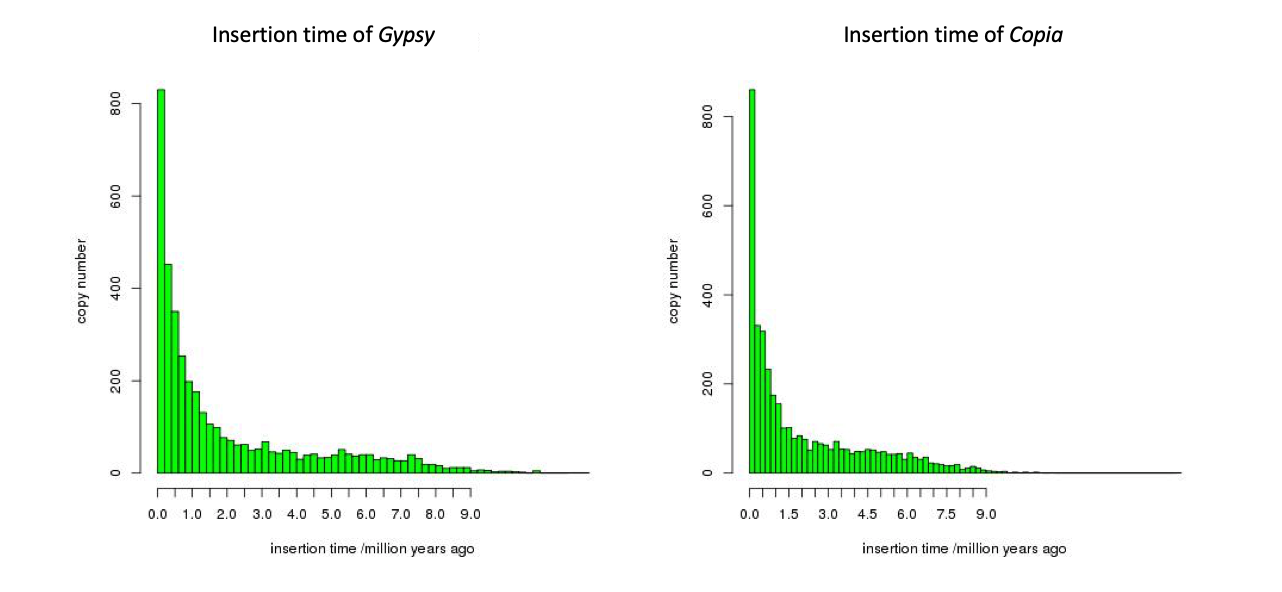


### Supplementary Fig. 4. Insertion burst of *Gypsy* and *Copia* retrotransposons in the *Actinidia eriantha* genome.


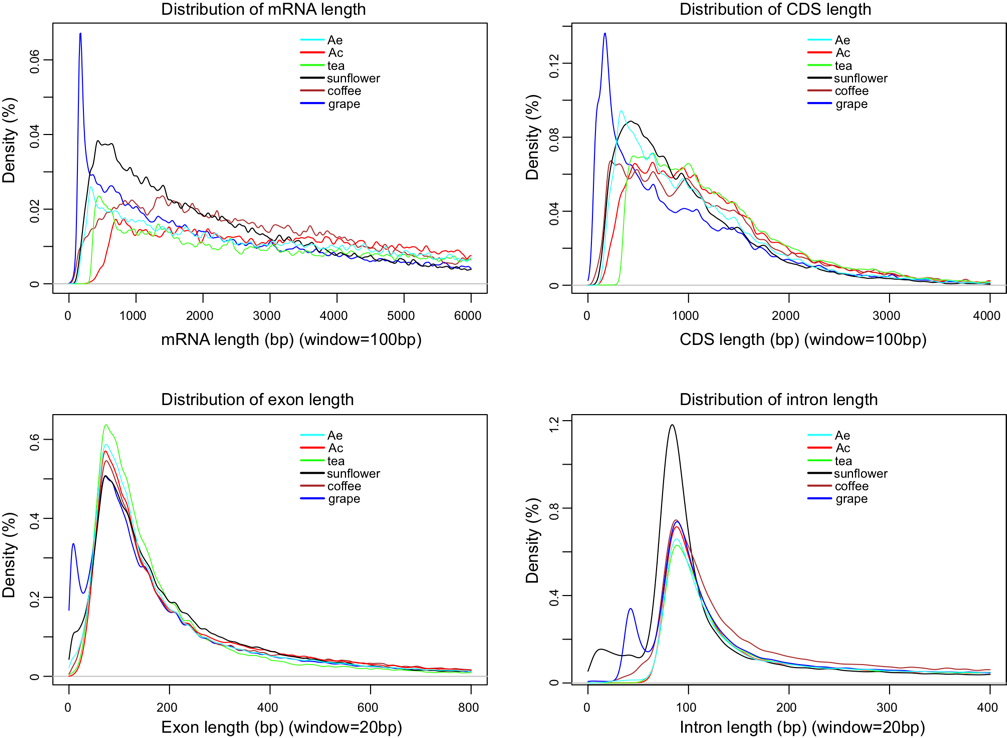


### Supplementary Fig. 5. The distribution of gene elements within six plant species. Ae: *Actinidia eriantha*, Ac: *A. chinensis*.


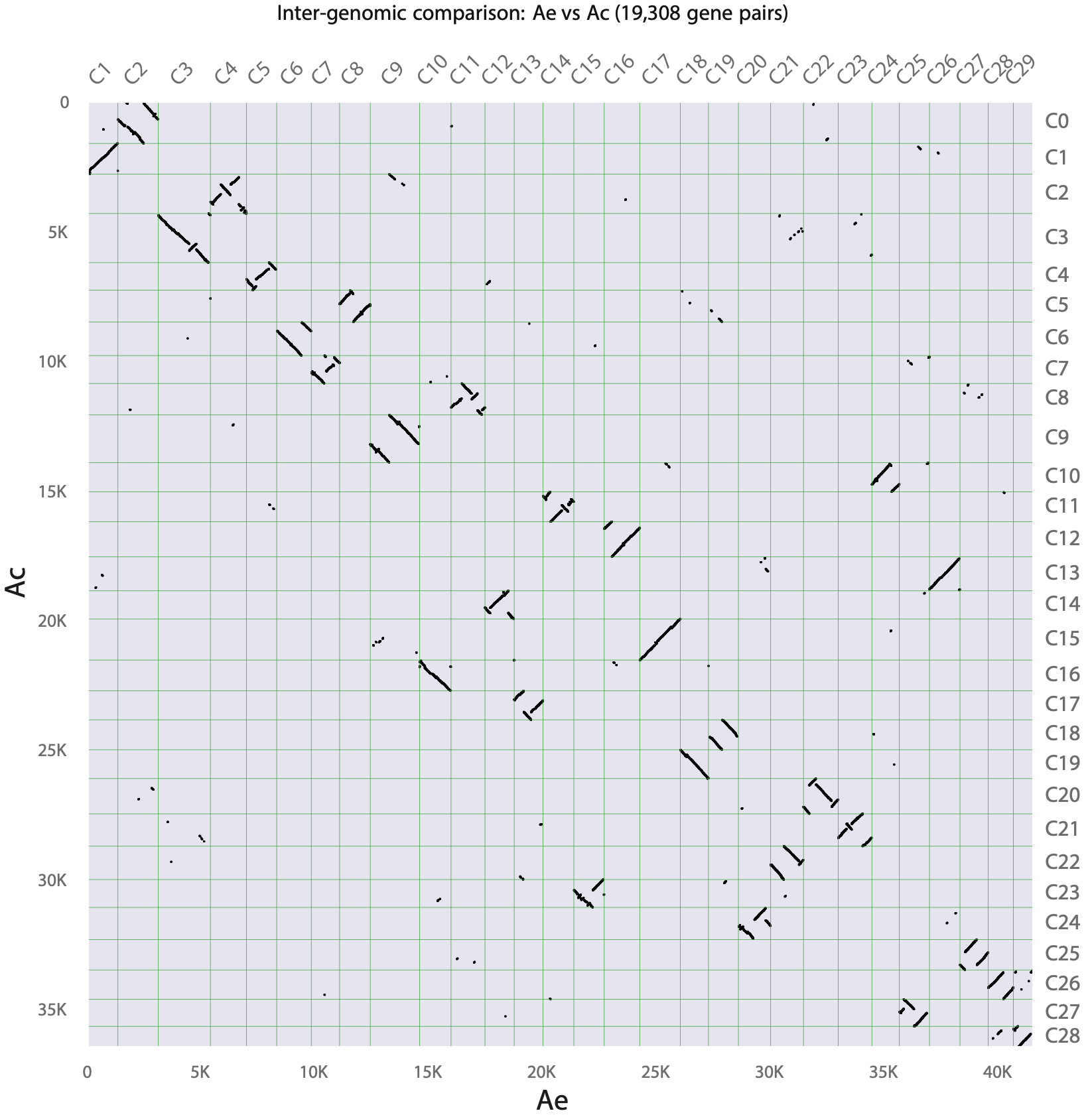


### Supplementary Fig. 6. The synteny between the *Actinidia eriantha* (Ae) and *A. chinensis* (Ac) pseudo-chromosomes.


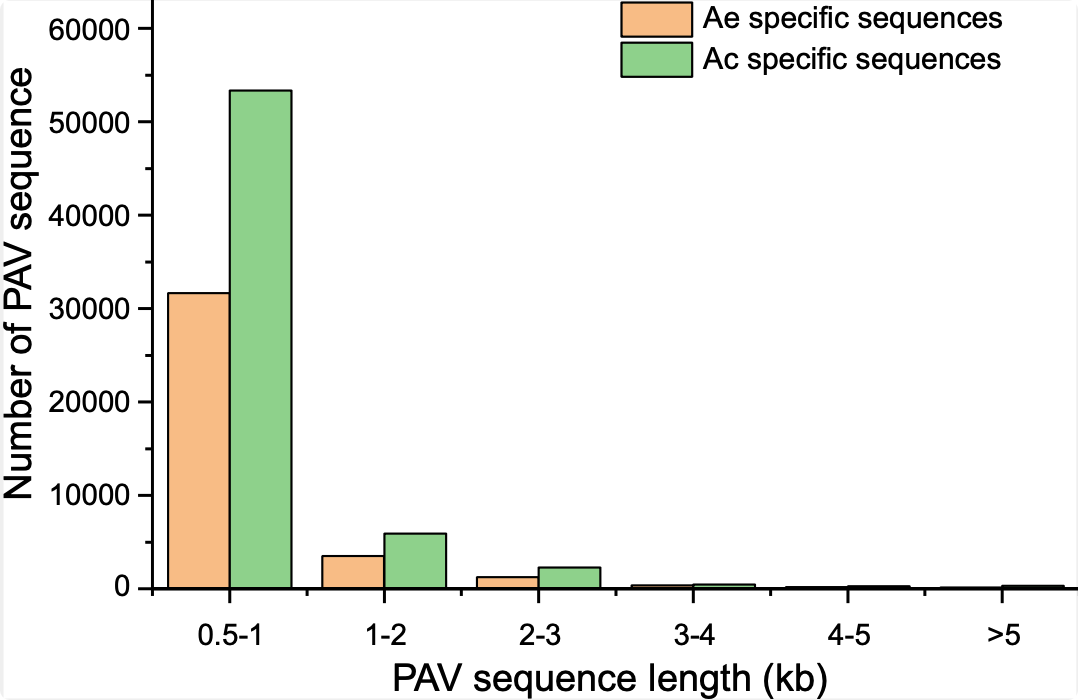


**Supplementary Fig.** **7.** The length distribution of presence/absence-variation (PAV) sequences in *Actinidia eriantha* (Ae) and *A. chinensis* (Ac).


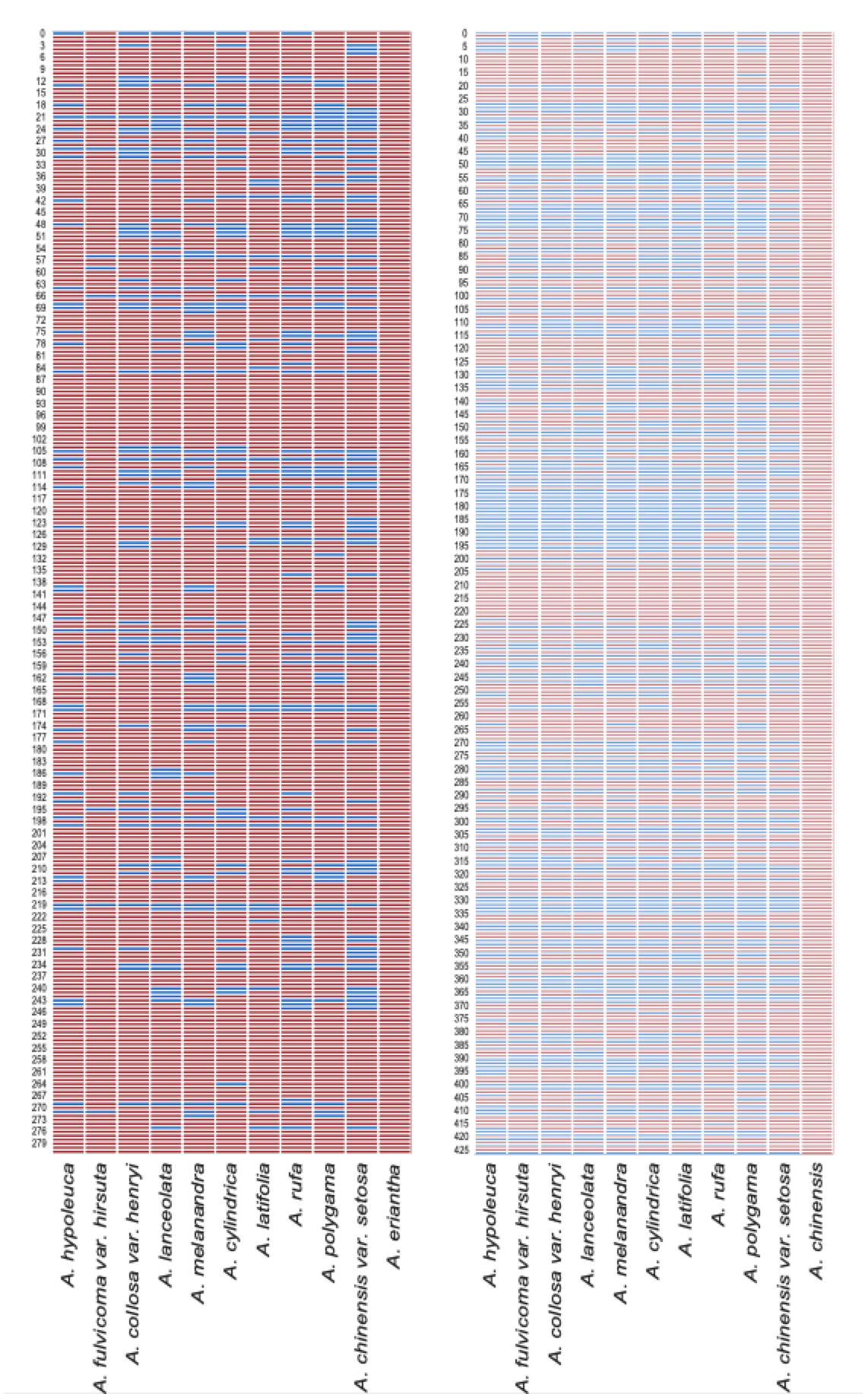


**Supplementary Fig.** **8.** Homologs of the *Actinidia eriantha*/*A. chinensis* presence/absence-variation genes across different *Actinidia* taxa.


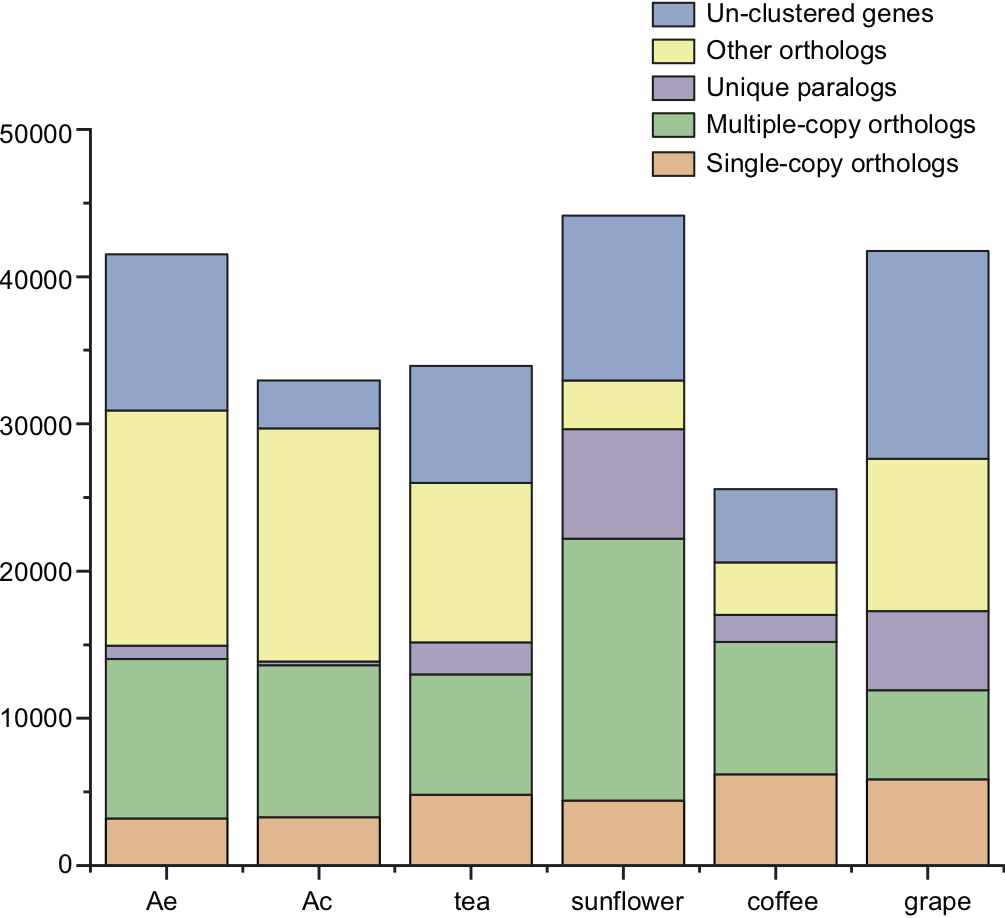


### Supplementary Fig. 9. Statistical analysis of orthologs or unique paralogs present in six plant species.


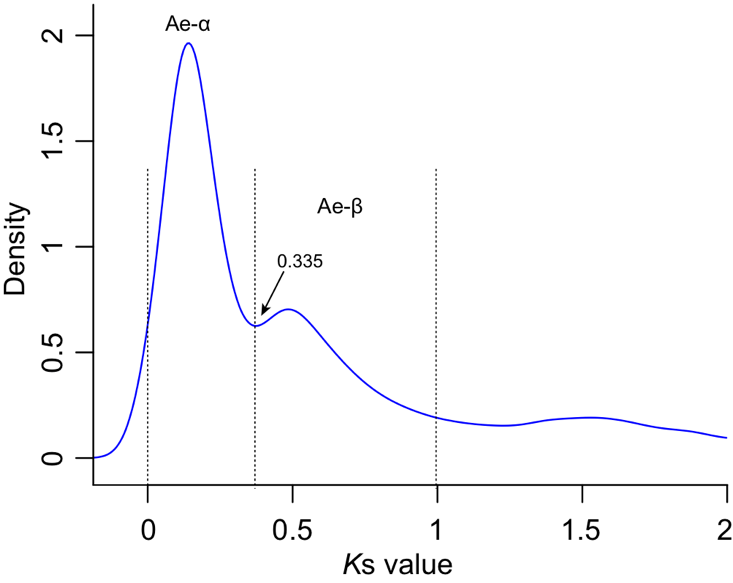


**Supplementary Fig.** **10.** Dividing Ae WGD events based on pairwise synonymous substitution rates (*K*_s_ values) of paralogs.


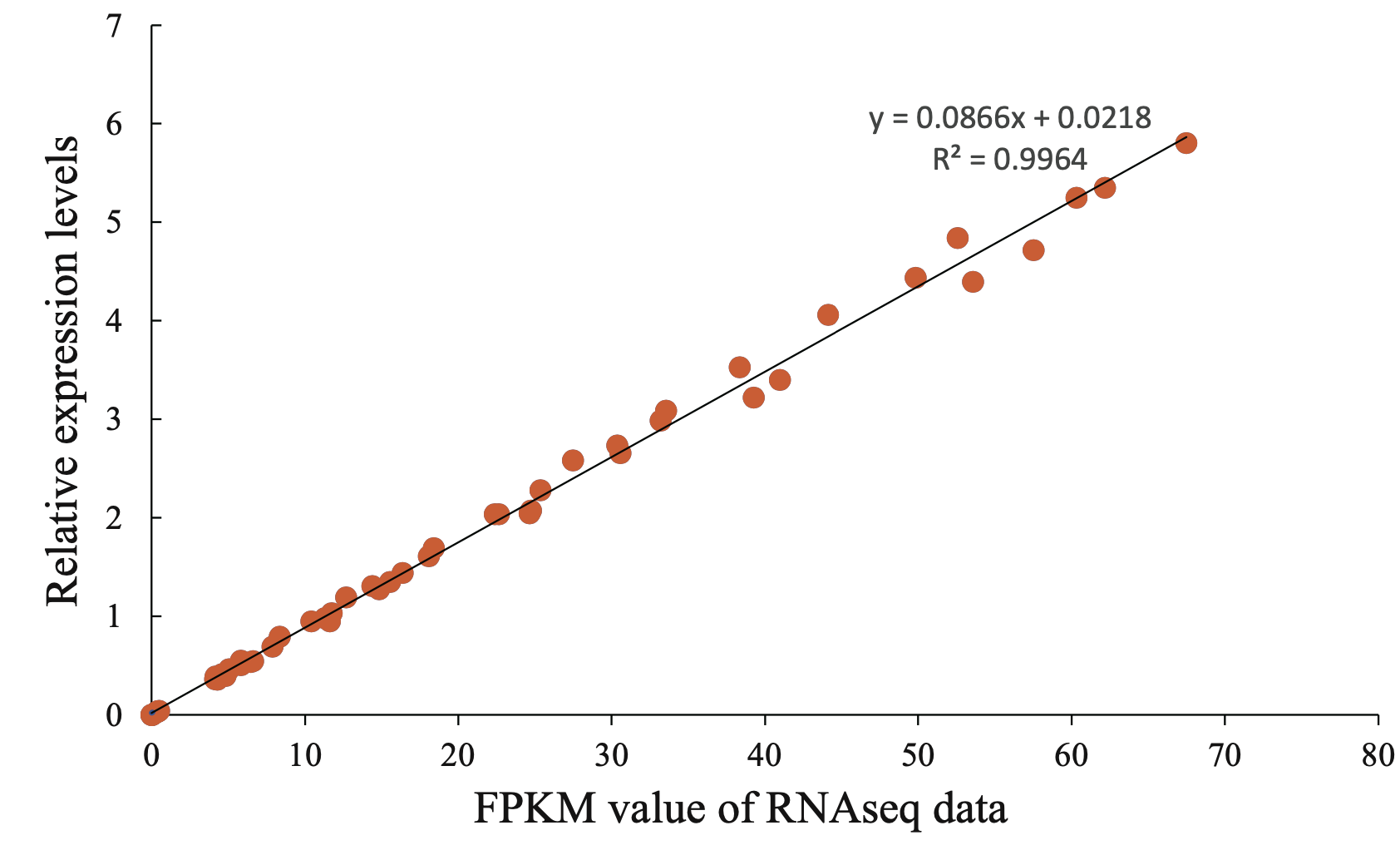


**Supplementary Fig. 11.** The consistent gene expressions in both transcriptome data and quantitative real-time polymerase chain reactions.


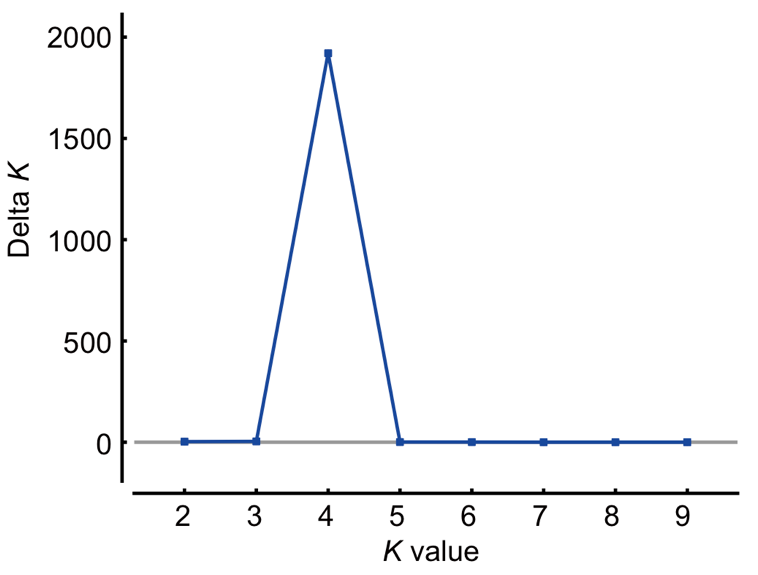


**Supplementary Fig.** **12.** The best *K* value estimated for STRUCTURE analysis of diverse kiwifruit taxa.

# Supplementary Tables

### Supplementary Table 1. Genome survey summary based on a *k*-mer analysis.

| **K-mer** | **K-mer number** | **K-mer depth** | **Genome size** | **Revised genome size** | **Heterozygous ratio** |
| --- | --- | --- | --- | --- | --- |
| 17 | 64,105,944,380 | 91 | 704.46 Mb | 689.07 Mb | 0.98% |

### Supplementary Table 2. Summary of the sequencing data of *Actinidia eriantha*.

| **Read type** | **Insert size** | **Total data (Gb)** | **Read length (bp)** | **Sequence coverage (****×)** |
| --- | --- | --- | --- | --- |
| Illumina reads | 350 bp | 79.54 | 150 | 116.97 |
| PacBio long reads | ­– | 60.13 | – | 88.42 |
| 10× Genomics | – | 105.30 | – | 154.85 |
| Total | – | 244.97 | – | 360.24 |

### Supplementary Table 3. Comparison of kiwifruit genome assembly statistics.

| **Statistic** | **Ae** | **Ac (v1)** | **Ac (v2)** | **Ac (v3)** | **Ac (Red5)** | **Ae (White)** |
| --- | --- | --- | --- | --- | --- | --- |
| Number of scaffolds | 580 | 7698 | 3887 | 2366 | 3887 | 1735 |
| Scaffold N50 | 5.07 Mb | 646.8 kb | 623.8 kb | 1.43 Mb | 567.7 kb | 23.58 Mb |
| Longest scaffold | 16.28 Mb | 3.41 Mb | 4.43 Mb | 7.81 Mb | 4.44 Mb | 28.6 Mb |
| Assembly size | 657.1 Mb | 616.1 Mb | 550.5 Mb | 653.9 Mb | 553.6 Mb | 690.6 Mb |

The kiwifruit genomes previously reported are collected from the KGD database (http://kiwifruitgenome.org/). Ae: *Actinidia eriantha*, Ac: *A. chinensis*.

### Supplementary Table 4. Summary of scaffolds in each chromosome-scale pseudomolecules.

| Pseudo-Chromosomes | Size (bp) | Scaffold number | Pseudo-Chromosomes | Size (bp) | Scaffold number |
| --- | --- | --- | --- | --- | --- |
| Chr01 | 22,575,243 | 10 | Chr16 | 27,233,501 | 15 |
| Chr02 | 19,699,266 | 9 | Chr17 | 20,509,276 | 4 |
| Chr03 | 27,194,696 | 16 | Chr18 | 24,063,825 | 9 |
| Chr04 | 14,992,323 | 9 | Chr19 | 24,909,023 | 8 |
| Chr05 | 22,775,714 | 12 | Chr20 | 20,656,410 | 8 |
| Chr06 | 21,378,665 | 7 | Chr21 | 18,643,001 | 6 |
| Chr07 | 23,897,141 | 19 | Chr22 | 22,297,304 | 12 |
| Chr08 | 27,634,844 | 17 | Chr23 | 22,793,972 | 11 |
| Chr09 | 17,746,071 | 3 | Chr24 | 19,718,081 | 6 |
| Chr10 | 22,452,265 | 12 | Chr25 | 23,068,820 | 13 |
| Chr11 | 18,397,420 | 10 | Chr26 | 19,741,079 | 9 |
| Chr12 | 22,934,038 | 15 | Chr27 | 23,776,980 | 12 |
| Chr13 | 20,745,460 | 8 | Chr28 | 18,772,926 | 10 |
| Chr14 | 21,549,331 | 12 | Chr29 | 20,238,136 | 6 |
| Chr15 | 19,425,810 | 6 | Total | 629,820,621 | 294 |

### Supplementary Table 5. Assessing *Actinidia eriantha* genome and annotation completeness with BUSCO analysis.

| **BUSCO notation** | **Number** | **Percent** |
| --- | --- | --- |
| Complete BUSCOs | 1342 | 93.2% |
| Complete and single-copy BUSCOS | 953 | 66.2% |
| Complete and duplicated BUSCOs | 389 | 27.0% |
| Fragmented BUSCOs | 21 | 1.5% |
| Missing BUSCOs | 77 | 5.3% |
| Total BUSCO groups searched | 1440 | 100% |

### Supplementary Table 6. Statistics of genomic repetitive contents of *Actinidia eriantha*.

| **Type** | **Repeat Size (bp)** | **% of genome** |
| --- | --- | --- |
| TRF | 28,226,441 | 4.30 |
| RepeatMasker | 251,150,253 | 38.22 |
| RepeatProteinMask | 70,893,334 | 10.79 |
| Total | 271,343,527 | 41.29 |

* TRF (tandem repeats finder) is a program to locate and display tandem repeats in DNA sequences. Both RepeatMasker and RepeatProteinMask are programs to screen DNA sequences for interspersed repeats and low complexity DNA sequences. Total is the combined result from all above three programs by removing redundant contents.

### Supplementary Table 7. Classification of the transposable elements (TEs) in the *Actinidia eriantha* genome.

| **Type** | **De novo + Repbase** | | | **TE Proteins** | | | **Combined TEs** | | |
| --- | --- | --- | --- | --- | --- | --- | --- | --- | --- |
|  | Length (bp) | % in genome | Length (bp) | | % in genome | Length (bp) | | % in genome |  |
| LTR | 190,053,467 | 28.92 | 62,740,908 | | 9.55 | 196,186,600 | | 29.86 |  |
| DNA | 37,480,303 | 5.70 | 4,518,220 | | 0.69 | 40,332,680 | | 6.14 |  |
| LINE | 5,709,531 | 0.87 | 3,771,832 | | 0.57 | 8,277,699 | | 1.26 |  |
| SINE | 239,794 | 0.04 | 0 | | 0.00 | 239,794 | | 0.04 |  |
| Unknown | 22,448,838 | 3.42 | 0 | | 0.00 | 22,448,838 | | 3.42 |  |
| Total | 251,150,253 | 38.22 | 70,893,334 | | 10.79 | 260,661,996 | | 39.67 |  |

### Supplementary Table 8. Summary of both the intact *Gypsy* and *Copia* LTR families.

| **Superfamily** | **Family** | **Number** | **Length (bp)** |
| --- | --- | --- | --- |
| *Gypsy* | *17_6* | 1 | 9,823 |
|  | *TF* | 12 | 55,539 |
|  | *athila* | 654 | 7,030,096 |
|  | *crm* | 906 | 4,720,808 |
|  | *del* | 1,590 | 14,502,986 |
|  | *galadriel* | 45 | 182,484 |
|  | *gypsy* | 1 | 7,886 |
|  | *pyret* | 1 | 8,888 |
|  | *reina* | 124 | 536,190 |
|  | *tat* | 671 | 7,126,972 |
| Total | - | 4,005 | 34,181,672 |
| *Copia* | *1731* | 8 | 30,675 |
|  | *copia* | 22 | 111,342 |
|  | *oryco* | 119 | 682,571 |
|  | *pseudovirus* | 4 | 31,732 |
|  | *retrofit* | 775 | 4,220,353 |
|  | *sire* | 1,004 | 9,952,444 |
|  | *tork* | 1,907 | 10,557,825 |
| Total | - | 3,839 | 25,586,942 |

### Supplementary Table 9. The identified transcription factor (TF) genes in the *Actinidia eriantha* genome.

| **TF family** | **Number** | **TF family** | **Number** | **TF family** | **Number** | **TF family** | **Number** |
| --- | --- | --- | --- | --- | --- | --- | --- |
| AP2 | 33 | CPP | 11 | LOB | 77 | S1Fa-like | 5 |
| ARF | 42 | CSD | 10 | M-type | 33 | SAP | 2 |
| ARR-B | 22 | DBB | 14 | MIKC | 57 | SBP | 38 |
| Alfin-like | 9 | DBP | 7 | MYB | 181 | SRS | 13 |
| B3 | 61 | E2F-DP | 14 | MYB-related | 123 | STAT | 1 |
| BBR-BPC | 19 | EIL | 8 | NAC | 164 | TCP | 41 |
| BES1 | 13 | ERF | 163 | NF-X1 | 2 | TUB | 19 |
| BSD | 1 | FAR1 | 6 | NF-YA | 16 | Tify | 19 |
| C2C2-CO-like | 8 | G2-like | 71 | NF-YB | 25 | Trihelix | 69 |
| C2C2-Dof | 53 | GRAS | 97 | NF-YC | 26 | ULT | 3 |
| C2C2-GATA | 49 | GRF | 25 | NOZZLE | 2 | VOZ | 5 |
| C2C2-LSD | 6 | GeBP | 13 | OFP | 37 | WRKY | 125 |
| C2C2-YABBY | 12 | HB | 152 | Orphans | 93 | Whirly | 3 |
| C2H2 | 176 | HRT | 1 | PLATZ | 21 | bHLH | 225 |
| C3H | 116 | HSF | 33 | RAV | 4 | bZIP | 109 |
| CAMTA | 10 | LFY | 2 | RWP-RK | 15 | zf-HD | 35 |

### Supplementary Table 10. Summary of aligned sequences, SNPs and Indels between Ae and Ac genomes.

|  | **Ae** | | **Ac v3** |
| --- | --- | --- | --- |
| Assembled genome size used for comparison | 657097116 | | 653926268 |
| One-to-one syntenic blocks | 394535301 | | 394408913 |
| Percentage of syntenic blocks in each genome | 60.0% | | 60.3% |
| SNPs | 15628085 | | |
| Insertions in Ae | 1814658 | | |
| Deletions in Ae | 1951635 | | |
|  | **Ae** | **Ae White** | |
| Assembled genome size used for comparison | 657097116 | 690781529 | |
| One-to-one syntenic blocks | 547350384 | 548420305 | |
| Percentage of syntenic blocks in each genome | 83.3% | 79.4% | |
| SNPs | 8181896 | | |
| Insertions in Ae | 3053384 | | |
| Deletions in Ae | 3563999 | | |

**Supplementary Table 11.** Enriched GO terms of the genes specific presented in our Ae genome. (in Supplemental_Excel_Tables.xlsx)

**Supplementary Table** **12.** Enriched GO terms of duplicated genes specific presented in both Ad-α and Ad-β events respectively. (in Supplemental_Excel_Tables.xlsx)

**Supplementary Table** **13.** Ascorbate-related genes investigated in both the *Actinidia eriantha* (Ae) and *A. chinensis* (Ac) genomes.

| **Enzyme name** | **Abbreviation** | **Gene number (Ae)** | **Gene number (Ac)** |
| --- | --- | --- | --- |
| Phosphoglucoisomerase | PGI | 3 | 3 |
| Phosphomannomutase | PMM | 2 | 1 |
| GDP-mannose pyrophosphorylase | GMP | 9 | 7 |
| GDP-mannose-3',5'-epimerase | GME | 3 | 3 |
| GDP-L-galactose phosphorylase | GGP | 2 | 3 |
| L-Galactose-1-phosphate phosphatase | GPP | 2 | 2 |
| L-Galactose dehydrogenase | GalDH | 13 | 16 |
| L-Galactono-1',4'-lactone dehydrogenase | GalLDH | 1 | 1 |
| L-Gulono-1,4-lactone oxidase/D- Arabinino-1,4-lactone oxidase | GuLO | 2 | 4 |
| GDP-D-mannose-4,6-dehydratase | MUR | 1 | 5 |
| GDP-L-fucosesynthase/GDP-4-keto-6-deoxy-D-mannose-3,5-epimerase-4-reductase | GER | 6 | 5 |
| UDP-glucuronate epimerase/UDP-galacturonate epimerase | UGlcAE/GAE | 14 | 15 |
| Polygalacturonate 4-α-galacturonosyltransferase | PGT | 44 | 36 |
| Pectin methylesterase | PME | 33 | 20 |
| Endopolygalacturonase | PG | 5 | 3 |
| D-Galacturonic acid reductase | GalUR | 28 | 16 |
| Aldonolactonase | Alase | 5 | 5 |
| L-Myo-inositol 1-phosphate synthase | MIPS | 1 | 2 |
| Myo-inositol oxygenase | MIOX | 8 | 6 |
| Phosphoglucomutase | PGM | 3 | 2 |
| UDP-glucose-pyrophosphorylase | UGP | 5 | 5 |
| UDP-glucose dehydrogenase | UGD | 4 | 5 |
| Glucuronate-1-phosphate uridylyltransferase | GluPU | 2 | 3 |
| Monodehydroascorbate reductase | MDAR | 5 | 8 |
| Dehydroascorbate reductase | DHAR | 3 | 4 |
| Protein kinase/protein phosphatase | VTC3 | 1 | 1 |
| Ethylene response factor subfamily b-3 of ERF/AP2 transcription factor family | ERF98 | 151 | 222 |
| Ascorbic acid mannose pathway regulator 1 (F box protein) | AMR1 | 12 | 8 |
| Cop9-signalosome 5b | CSN5B | 4 | 5 |
| Constitutive photomorphogenic 9 | CSN8 | 4 | 2 |
| Anion transporter 2 | ATPHT4 | 10 | 11 |

**Supplementary Table 14.** Primers used for quantitative real-time polymerase chain reaction analysis.

| **Genes** | | **Primer name** | **Sequences (5'->3')** |
| --- | --- | --- | --- |
| Ascorbic acid biosynthesis | PGT | scaf_18.319-F | GGTGAACGGAGCTGTTGAGA |
|  |  | scaf_18.319-R | ATTGTACCCGAGTCCGAGGA |
|  |  | scaf_112.80-F | AGAGCAGCATCTTTCGCCTT |
|  |  | scaf_112.80-R | GATCCAATCGCGGCTTCAAC |
|  | PG | scaf_32.130-F | ACCGATGGGATCCACCTACA |
|  |  | scaf_32.130-R | ATTCCTGTTAGGGCCACAGC |
|  |  | scaf_66.371-F | CCGCATTCGATGACCCCATA |
|  |  | scaf_66.371-R | TAGGTGGATCCCATCGGTGT |
|  | GaIUR | scaf_38.347-F | CATCAACGCCATCAAGCTCG |
|  |  | scaf_38.347-R | CCCAAAGCGCTTCCAGTTTC |
|  |  | scaf_66.301-F | CCCGGTAAATACGAGGCTCC |
|  |  | scaf_66.301-R | CCCCTTGCTCATGAACCCAT |
|  | PME | scaf_86.150-F | TAGTTTGATTGACCCGGCCG |
|  |  | scaf_86.150-R | TCAAAGGCCTGACGTGAAGG |
|  |  | scaf_123.120-F | TGTCCTCACCGGTGTCATTG |
|  |  | scaf_123.120-R | TCGCAATTCTGGAGGACCAC |
| Disease-resistance | | scaf_65.124-F | GACTTCAGACCCAGACGCAA |
|  |  | scaf_65.124-R | ATCCACATCCGCATCGAGTC |
|  |  | scaf_66.392-F | GCTTGGTGGACGAGTGGTAA |
|  |  | scaf_66.392-R | ATTCCGCCCATTCCCCAAAT |
|  |  | scaf_122.355-F | CCAATCTCCTCCAGCAGGTG |
|  |  | scaf_122.355-R | TGTGCGGTGCTACTCAAGAG |
| Inner standard | | AeActin-PF | GCTTACAGAGGCACCACTCAACC |
|  |  | AeActin-PR | CCGGAATCCAGCACCAATACCAG |

**Supplementary Table** **15.** Nucleotide-binding site (NBS) genes identified in both *Actinidia eriantha* (Ae) and *A. chinensis* (Ac) genomes.

| **Category** | **Ae** | **Ac** |
| --- | --- | --- |
| CC-NBS-LRR | 15 | 22 |
| CC-NBS | 11 | 9 |
| TIR-NBS-LRR | 5 | 7 |
| RPW8-NBS-LRR | 2 | 2 |
| NBS-LRR | 20 | 59 |
| TIR-NBS | 3 | 7 |
| others | 39 | 33 |
| Total | 95 | 139 |

**Supplementary Table** **16.** Disease resistance genes specially expressed in *Actinidia eriantha* (Ae) and *A. chinensis* (Ac) respectively.

| **Gene ID** | **Expression levels （FPKM）** | | | | | | | | | | | | **Annotation** |
| --- | --- | --- | --- | --- | --- | --- | --- | --- | --- | --- | --- | --- | --- |
|  | **Ac-1-1** | **Ac-2-1** | **Ac-1-2** | **Ac-2-2** | **Ac-1-3** | **Ac-2-3** | **Ae-1-1** | **Ae-2-1** | **Ae-1-2** | **Ae-2-2** | **Ae-1-3** | **Ae-2-3** |  |
| scaf_78.334 | 0 | 0 | 0 | 0 | 0 | 0 | 1.387 | 1.001 | 4.065 | 1.875 | 1.545 | 7.319 | putative late blight resistance protein homolog R1A-10 |
| scaf_105.241 | 0 | 0 | 2.242 | 0 | 0 | 0.687 | 2.78 | 0.978 | 1.238 | 1.926 | 3.571 | 0.954 | TMV resistance protein N-like |
| scaf_105.262 | 0 | 0 | 0 | 0 | 0 | 0 | 5.581 | 4.725 | 4.672 | 7.188 | 5.87 | 4.832 | disease resistance protein RPS2-like |
| scaf_185.172 | 0 | 0 | 0 | 0 | 0 | 0 | 8.255 | 7.312 | 14.695 | 8.801 | 12.5 | 13.645 | putative disease resistance protein At1g50180 isoform X1 [Vitis vinifera] |
| scaf_58.60 | 0 | 0 | 0.867 | 0 | 0 | 0 | 3.014 | 3.277 | 3.647 | 3.859 | 4.276 | 2.956 | putative disease resistance protein RGA3 |
| scaf_122.289 | 0.575 | 0.49 | 0.718 | 0.681 | 0.405 | 0.389 | 0 | 0 | 0 | 0 | 0 | 0 | hypothetical protein CICLE_v10020011mg [Citrus clementina] |
| scaf_169.145 | 0.599 | 0.453 | 5.957 | 0.483 | 0.831 | 0.326 | 0 | 0 | 0 | 0 | 0 | 0 | NB-ARC domain-containing disease resistance protein, putative isoform 1; K13459 disease resistance protein RPS2 (A) |
| scaf_1.235 | 1.055 | 1.711 | 6.168 | 2.141 | 3.537 | 1.811 | 0 | 0 | 0 | 0 | 0 | 0 | TMV resistance protein N, putative; K19613 leucine-rich repeat protein SHOC2 (A) |
| scaf_65.124 | 13.846 | 11.644 | 18.479 | 14.378 | 17.268 | 14.962 | 0 | 0.038 | 0.041 | 0.036 | 0 | 0 | putative disease resistance RPP13-like protein 3; K13457 disease resistance protein RPM1 (A) |
| scaf_111.962 | 0.252 | 0.509 | 1.907 | 1.163 | 1.747 | 1.728 | 0 | 0 | 0 | 0.094 | 0 | 0 | LRR receptor-like kinase family protein; K17422 large subunit ribosomal protein L41 (A) |
| scaf_66.207 | 5.191 | 8 | 25.997 | 13.785 | 3.217 | 5.906 | 0 | 0 | 0 | 0 | 0 | 0 | probable disease resistance protein At4g27220; K13459 disease resistance protein RPS2 (A) |

### Supplementary Table 17. List of *Actinidia* taxa used for transcriptomic sequencing.

| **Fruit skin type** | **Abbreviation** | **Taxon** |
| --- | --- | --- |
| SHS (soft and hairless skins) | KOL | *A. kolomikta* |
|  | ARG1 | *A. arguta* |
|  | ARG2 | *A. arguta* |
|  | ARG3 | *A. arguta* |
|  | MAC | *A. macrosperma* |
|  | VAL | *A. valvata* |
|  | POL | *A. polygama* |
| RWS (rough and warty skins) | RUF | *A. rufa* |
|  | IND1 | *A. indochinensis* |
|  | IND2 | *A. indochinensis* |
|  | IND3 | *A. indochinensis* |
|  | CHI | *A. chinensis* |
|  | CYL | *A. cylindrica* |
|  | HEN | *A. callosa* var. *henryi* |
| RHS (rough and hairy skins) | DEL | *A. chinensis* var. *deliciosa* |
|  | ERI1 | *A. eriantha* |
|  | ERI2 | *A. eriantha* |
|  | ERI3 | *A. eriantha* |
|  | FUL | *A. fulvicoma* |
|  | RET | *A. cylindrica* var. *reticulata* |
|  | STY | *A. styracifolia* |
